# Canadian National Pancreas Conference 2025: Recent Advances in Patient-Centred Management of Pancreatic Cancer

**DOI:** 10.3390/curroncol33100629

**Published:** 2026-10-19

**Authors:** Jessica L. Nickerson, Stefanie Condon-Oldreive, Erica Tsang, George Zogopoulos, Rodney Ouellette, Orçun Haçariz, Beatriz Antón Pascual, Pablo E. Serrano, Sharlene Gill, Saman Maleki Vareki, Louise Binder, Leah Stephenson, Breffni Hannon, Jaymee Maaghop, Popi Kasvis, Ken Leslie

**Affiliations:** 1 Craig’s Cause Pancreatic Cancer Society, Halifax, NS B3K 5M3, Canada; jessica@craigscause.ca (J.L.N.); stefanie@craigscause.ca (S.C.-O.); 2 Department of Medical Oncology, Princess Margaret Cancer Centre, Toronto, ON M5G 2C4, Canada; erica.tsang@uhn.ca (E.T.); beatriz.antonpascual@uhn.ca (B.A.P.); 3 Departments of Surgery and Oncology, McGill University and the McGill University Health Centre, Montreal, QC H4A 3J1, Canada; george.zogopoulos@mcgill.ca; 4 Atlantic Cancer Research Institute, Moncton, NB E1C 8X3, Canada; rodneyo@canceratl.ca; 5 Department of Surgery, Division of Surgical and Interventional Sciences, McGill University, Montreal, QC H4A 3J1, Canada; orcun.hacariz@mail.mcgill.ca; 6 Cancer Research Programme, Research Institute of the McGill University Health Center, Monteal, QC H4A 3J1, Canada; 7 Department of Surgery, Juravinski Hospital and Cancer Centre, Hamilton, ON L8V 1C3, Canada; serrano@mcmaster.ca; 8 BC Cancer, Vancouver, BC V5Z 4E6, Canada; sgill@bccancer.ca; 9 Departments of Pathology and Laboratory Medicine, Western University, London, ON N6A 3K7, Canada; saman.malekivareki@lhsc.on.ca; 10 Department of Oncology, Western University, London, ON N6A 3K7, Canada; 11 Save Your Skin Foundation, Pentincton, BC V2A 0B2, Canada; louise.binder49@gmail.com; 12 All.Can Canada, Pentincton, BC V2A 0B2, Canada; leah.allcancanada@saveyourskin.ca; 13 Department of Supportive Care, Princess Margaret Cancer Centre, Toronto, ON M5G 2C4, Canada; breffni.hannon@uhn.ca; 14 Gastrointestinal Society, Vancouver, BC V5R 5W2, Canada; 15 School of Human Nutrition, McGill University, Montreal, QC H9X 3V9, Canada; kalliopi.kasvis@mcgill.ca; 16 Baker Centre for Pancreatic Cancer, London Health Sciences Centre, London, ON N6A 5W9, Canada

**Keywords:** pancreatic cancer, multidisciplinary care, patient-centred care, genomic sequencing, clinical trials, pancreatic enzyme replacement therapy, drug funding, national guidelines

## Abstract

Pancreatic cancer (PC) is among the most lethal malignancies with slower progress in overall survival and treatment options compared to other cancers. The 2025 Canadian National Pancreas Conference (NPC)—the third biennial event of its kind—was hosted by Craig’s Cause Pancreatic Cancer Society and the Baker Centre for Pancreatic Cancer in November 2025 in London, Ontario, co-organized by the Canadian Association of General Surgeons (CAGS) with accreditation through the Royal College of Physicians and Surgeons of Canada. The event brought together experts across healthcare disciplines as well as researchers, students, and allied professionals to discuss the latest advances in treatment and management of this complex disease. The conference featured presentations, workshops, and discussions on the current landscape of treatment and management of PC with an emphasis on the increasing significance of genomic sequencing, equitable care, and a multidisciplinary approach.

## Simple Summary

Pancreatic cancer is one of the leading causes of cancer-related death, yet progress in treatment and management has been slower compared to other cancers. The 2025 National Pancreas Conference brought together experts in pancreatic cancer across healthcare, research, and supportive care disciplines to discuss recent advances and next steps in treatment, management, research, and drug access. The present review summarizes the key findings and next steps proposed from the conference.

## 1. Introduction

In 2025, pancreatic cancer (PC) remains among the most lethal types of cancers, accounting for 3% of cancer cases and 8% of cancer-related deaths [1]. While survival rates for breast, cervical, gastric, and prostate cancers have made a range of progress over the last 50 years, the five-year survival for PC holds steady at 10% [2]. However, recent work demonstrated a doubling in overall survival for patients with metastatic pancreatic ductal adenocarcinoma (PDAC) using daraxonrasib in the second line [3]. This represents the most promising new treatment for metastatic PDAC in decades.

The relatively asymptomatic progression of PC poses a challenge in achieving early diagnosis, with additional disparities in outcomes noted based on race and socioeconomic status [4]. Eligibility for uniquely curative resection relies on early diagnosis, motivating effective screening, minimized wait times, and efficient detection. Currently, only 10–20% of patients are diagnosed early enough for their disease to be considered resectable [5], contributing significantly to the low survival rates. The challenges associated with early detection emphasize the importance of effective treatment and management through later stages of disease progression [6]. The adoption of inter-professional supportive care has proven effective in improving quality of life and overall survival (OS) [7,8].

The 2025 National Pancreas Conference (NPC)—hosted by Craig’s Cause Pancreatic Cancer Society and the Baker Centre for Pancreatic Cancer in November 2025 in London, Ontario—aimed to connect experts across PC care and research disciplines to discuss the advances in detection, treatment, and management since the 2023 NPC event [9] towards the elucidation of current best practices. The event was co-developed by the Canadian Association of General Surgeons (CAGS) and received accreditation through the Royal College of Physicians and Surgeons, enabling healthcare providers to earn Section I Maintenance of Certification credits. The event hosted diverse experts including oncologists, surgeons, researchers, dietitians, medical residents, and patient advocates. Session topics were defined following the results of a needs assessment including a review of recent literature and a survey of the target audience. Particular interest was expressed in neoadjuvant therapy, genomic sequencing, and immunotherapy. The results of the needs assessment are summarized in Supplementary File S1. It is noted that the accuracy of the survey results, in terms of representing the target audience, is limited by the volume of voluntary responses (25), with a bias towards surgeons (42%), medical oncologists (21%), and researchers (17%), with 84% of respondents working in an urban setting. The complete conference programme is shared as Supplementary File S2.

The increasing importance of accessible and timely genomic sequencing emerged as a connecting theme across session topics with benefits in prognostication, uptake of targeted therapies, as well as prediction of treatment response. The value of multidisciplinary patient-centred care was also highlighted with guidance on how to implement such models and lessons learned during the development of existing centres. The conclusions of the 2025 NPC are anticipated to guide the development of national guidelines on current best practices for 1) diagnosis and screening, 2) treatment and management of PC, and 3) utilization of pancreatic enzyme replacement therapy (PERT). The present review summarizes discussions and next steps concluded from the 2025 National Pancreas Conference, and is contextualized by relevant literature published prior to as well as following the NPC event.

## 2. Proven Models, National Impact: Patient-Centred Care

The high frequency of late-stage diagnosis and variable response to treatment in PC motivate a multidisciplinary and patient-centred approach to treatment and management, where access to surgeons, oncologists, clinical trial education, nutrition and PERT, genetic counselling, and more is facilitated. The benefits of multidisciplinary care in PC span improved overall survival, quality of life, reduced wait times for assessment, diagnosis, and symptom management, and more frequent interactions with the supportive care team [10]. In Canada, the Baker Centre for Pancreatic Cancer (London Health Sciences Centre, ON) and the Wallace McCain Centre for Pancreatic Cancer (Princess Margaret Cancer Centre, ON) epitomize this multidisciplinary care model—the logistics of their programmes were discussed while encouraging other provinces to adopt similar models.

**Figure 1:**
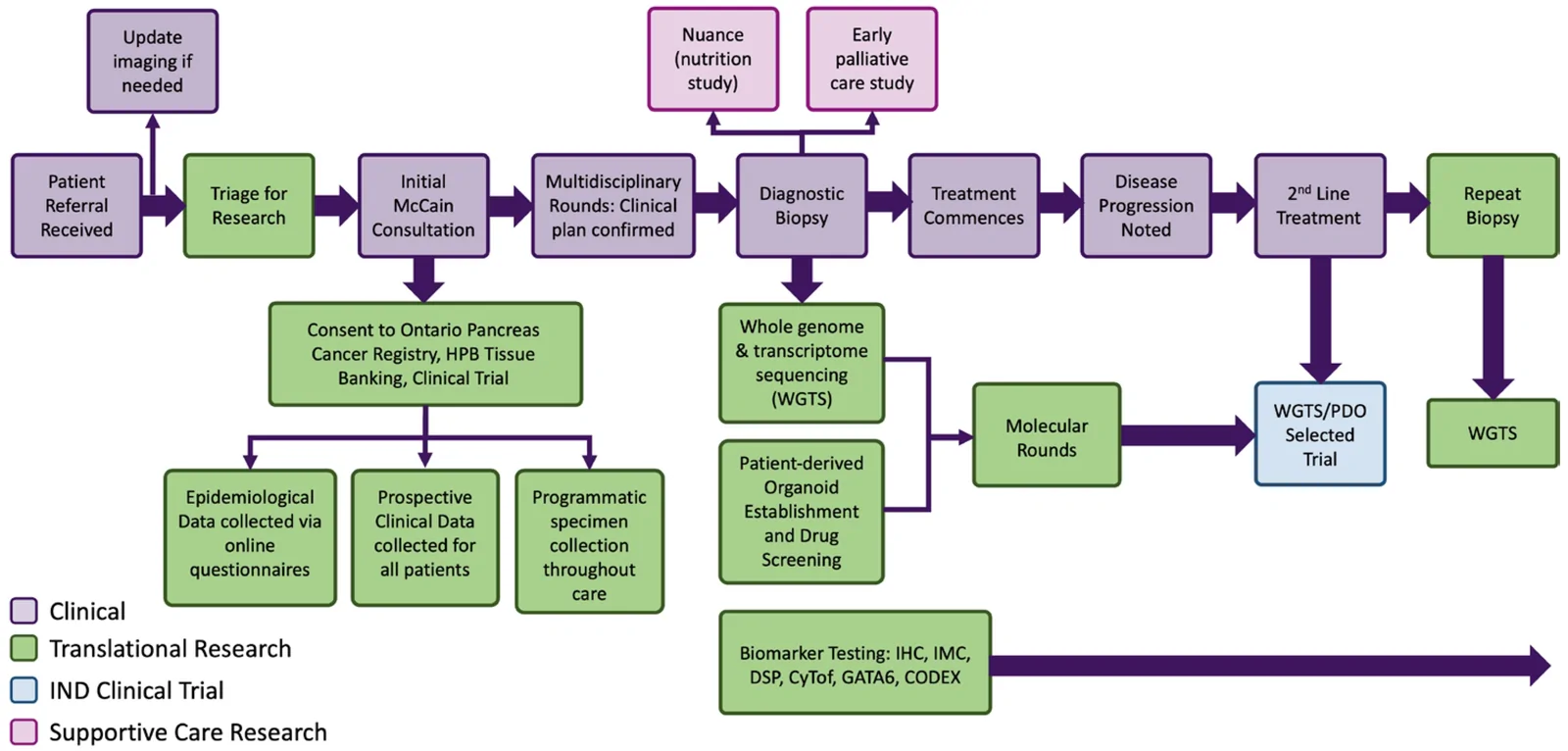
Multidisciplinary care pathway employed by the Wallace McCain Centre for Pancreatic Cancer (Toronto, ON, Canada) facilitating patient-centred care and frequent participation in research.

The Baker Centre’s programme was described to rely heavily on a nurse navigator, who serves as a central touchpoint for the patient and their multidisciplinary care team. The nurse navigator at the Baker Centre currently is in constant communication with 400–450 patients annually, whereby she triages their referrals to surgery, ensures their preparation for subsequent appointments, prioritizes cases for weekly discussion, among other important tasks. The expedited care delivered by both, the Baker Centre and Wallace McCain Centre, is achieved in part by encouraging referrals for patients who do not yet have a tissue-confirmed diagnosis; the cost associated with seeing a small fraction of benign cases has been outweighed by the benefit to the majority representing late-stage pancreas cancer patients. The Wallace McCain Centre efficiently leverages the first biopsy for both diagnosis and research with 74% of patients participating in research. Furthermore, integration of clinical and translational research facilitates many trials including those working on genomic profiling (COMPASS), personalized therapies (ADOPT, MOHCCN), nutrition support (Nuance), and access to treatments (Prosper-PANC). The multidisciplinary care model starts at the patient’s first appointment where they meet with oncology, surgery, the nurse navigator, a social worker, a dietitian, and researchers. Supportive care for PC relies heavily on effective nutrition and pancreatic enzyme replacement therapy. The Baker Centre team features a full-time dietitian who aims to see all PC patients with follow-ups as needed. The Wallace McCain Centre has all patients screened by a social worker and genetic counsellor as well. Figure 1 depicts the care pathway employed by the Wallace McCain Centre.

## 3. Genome Sequencing and Molecular Profiling

Effective treatment and management of PC is challenged by the high frequency of late diagnosis and treatment resistance. While mortality rates for other cancers show declining trends over the last several decades, little improvement has been seen for PC survival [11]. Survival rates are proposed to improve by (1) increasing the frequency of early diagnosis and (2) developing better biomarker-informed treatments for a more personalized approach. Recent progress in genomic sequencing and individualized treatment was discussed.

Novel genomic and transcriptomic biomarker signatures show promise for improving rates of early detection [12,13]. An early trial for targeted treatment of PC was published by Moore et al. in 2007 [14], wherein HER1/EGFR genes were targeted by combining erlotinib with the standard gemcitabine treatment. The 10-day improvement in survival was statistically significant but left much to be desired in terms of clinical/practical impact. Since then, advancements out of the Human Genome Project have drastically improved our access to an individual’s biology [15,16,17,18,19]. We have since learned that approximately 90% of PDAC cases are driven by a KRAS mutation and have gained insight on how this mutation progresses from precursor lesions to PDAC cells [20]. In the cases of wildtype KRAS, there is often another targetable driver such as fusions of FGFR2, NRG-1, NTRK, or RET. This deeper understanding of individual tumour development/progression helps with both prognosis and treatment. The COMPASS trial, published in 2025 [21], performed whole genome and transcriptome sequencing on 217 PC patients prior to standard treatment with either modified FOLFIRINOX or Gemcitabine nab-paclitaxel. Differential overall survival based on the biology of the PC cells revealed several prognostic groups. High frequency of a particular single base substitution profile, SBS3, and high HRDetect score (>0.7) correlated with better overall survival (OS) while basal-like and systemic inflammation correlated with worse OS in patients with advanced PDAC following standard treatments. The results of this important trial invite continued use of whole genome/transcriptome profiling when elucidating an individual’s course of treatment.

Personalized treatment may be approached by either germline (hereditary) or somatic (specific to the tumour) genetic sequencing. While germline testing offers the benefit of informing a patient’s relatives of their hereditary risk, somatic testing enables optimized treatment selection based on the tumour’s molecular profile. The MSK-IMPACT trial demonstrated that at least one of the 100 profiled cancer-related genes had a mutation in 17% of the evaluated cancer patients, which supports the adoption of germline genetic sequencing for all patients [22]. Specifically, patients harbouring a BRCA/PALB2 mutation have been shown to benefit from platinum-based therapy with a maintenance PARP inhibitor—Golan et al. found an improvement in progression-free survival (7.4 months vs. 3.8 months (*p* = 0.004)), albeit no significant difference in overall survival [23]. More recent recommendations from the National Comprehensive Cancer Network recommend germline as well as somatic testing [24], given 20–30% of tumours exhibit actionable mutations and the relevance for clinical trial eligibility. Somatic testing should emphasize microsatellite instability, a mismatch repair gene panel, and RNA sequencing for gene fusions. Table 1 summarizes several results from somatic profiling and their respective optimal course of treatment. Patients with no currently targetable mutation are encouraged to participate in a clinical trial, while ongoing work aims to optimize KRAS-directed therapy.

## 4. Nutrition and Pancreatic Enzyme Replacement Therapy (PERT)

Nutrition management is a key challenge and prognostic indicator in PC owing to side effects such as abdominal pain and pancreatic exocrine insufficiency (PEI) resulting in low appetite and malabsorption. 80% of patients report unexplained weight loss at the time of diagnosis, driven by systemic metabolic mechanisms including increased rates of lipolysis in adipose tissue, malabsorption of fat from food, and systemic inflammation leading to sarcopenia (muscle wasting) [25]. Despite the prevalence of poor nutrition, only 25% of PC patients receive nutritional support [26]. A recent review by Ponz de Leon Pisani et al. summarized the complex effects of PC on the gastrointestinal tract [27], revealing several ailments that may be targeted by dietitians: anorexia/cachexia, nausea/vomiting, and diarrhea/steatorrhea. The current recommendation is that all PC patients should be seen by a dietitian [28]. Diagnosis of PEI, cachexia, and sarcopenia is critical for effective nutrition management, which may be facilitated by exploiting the diagnostic CT scan to determine body composition [29]. PEI in PC is suspected in the presence of weight loss and/or signs and symptoms of PEI. Treatment should not be delayed in the absence of a formal diagnosis. PEI may be formally diagnosed via a fecal elastase test; however, it is more practically diagnosed based on an assessment of symptoms (nutritional status, apparent pancreatic function) [30,31]. If PEI is suspected, a trial of pancreatic enzyme replacement therapy (PERT) is recommended, with evidence showing improved OS associated with PERT usage following a retrospective study on patients with unresectable PC [32]. The recommended dosage varies but should be titrated by each patient based on signs and symptoms of PEI. Uptake of PERT is further challenged by accessibility, which may be improved by enabling registered dietitians to prescribe PERT as well as by addressing shortages. Alternatively, patients may benefit from digital resources such as the PERT Calculator, a tool to assist with PERT dosage based on input meals or snacks and individual information especially when access to a dietitian is limited [33]. Patients describe rationing capsules due to shortages and/or to reduce out-of-pocket cost—provinces are implored to conduct a deeper cost/outcome analysis of administering chemotherapy, radiation, and/or surgery if nutrition is poorly managed and subsequently revisit the favourability of universal coverage for PERT.

## 5. Research and Clinical Trials

The number of treatment options for PC has lagged compared to other cancers owing to late diagnosis, the chemotherapy-resistant tumour microenvironment (dense/fibrous stroma), and low immunogenicity [34]. However, advances in molecular profiling and immunotherapy are paving new avenues for advanced PC treatment and treatment selection. FOLFIRINOX has previously been associated with higher toxicity than Gemcitabine nab-Paclitaxel [35]; however, a retrospective study suggested more favourable outcomes from FOLFIRINOX [36]. A randomized trial would be required to make a more definitive comparison between the two approaches. The NAPOLI-3 trial compared a regimen of liposomal irinotecan + 5-fluorouracil/leucovorin + oxaliplatin (NALIRIFOX) to Gem nab-Paclitaxel as first-line treatment for metastatic PC. NALIRIFOX demonstrated a 2-month improvement in median OS and progression-free survival. Despite the improvements in patient outcomes, uptake of NALIRIFOX is limited by its high cost [37]; discussions questioned whether the benefit outweighed the cost. The COMPASS and PASS-01 trials characterized the genetic mutations predictive of response to FOLFIRINOX vs. Gemcitabine nab-Paclitaxel [21]. Specifically, a high HRDetect score was found to correlate with better OS on modified FOLFIRINOX compared to Gemcitabine nab-Paclitaxel.

Expanding outside of the two standard drugs, several trials are striving towards KRAS-targeted approaches in hopes of offering a targeted therapy applicable to the majority of PDAC patients. A KRAS G12C mutation (present in 1–2% of PC cases) is targeted by inhibitors Sotorasib and Adagrasib in CodeBreaK100 [38] and KRYSTAL-1 trials [39], respectively. To date, both drugs demonstrate anti-cancer activity and safety. Zoldonrasib (RMC-9805) shows promise as a KRAS G12D inhibitor in mouse models [40]. A 2025 study by Bandi et al. describes a more widely applicable approach, where ADT-1004 demonstrated allele-agnostic RAS inhibition in a pre-clinical setting [41]. Despite the ongoing progress in developing KRAS inhibitors [42], uptake is impeded by limited access in Canada.

Novel immunotherapy approaches are central to recent clinical trials across solid tumour malignancies, however PC is plagued by low immune cell infiltration and an immunosuppressive microenvironment [43,44]. Recent work shows promise in combining immunotherapy with chemotherapy [45,46]. PC is notorious for exhibiting variable response to treatment. Many current trials and foundational research studies focus on making immunotherapy approaches accessible and effective for PC patients. The role of the tumour microbiome in response to treatment was discussed. Enhanced T cell infiltration was demonstrated in longer-term survivors compared to short-term survivors, as well as the presence of microbial neoantigens that elicit an immune response which coincidentally reduces the tumour [47]. In 2017, Riquelme et al. defined a specific microbiome signature correlative with longer survival, which may have prognostic benefits [48]. In addition, gammaproteobacteria (commonly present in PDAC malignancies) was found to metabolize Gemcitabine while the resulting metabolites promote T cell exclusion [49]. Given the apparent influence of the tumour microbiome on immunotherapy efficacy, modulation of the tumour microbiome by way of fecal microbiota transplantation (FMT) has emerged [50,51,52]. Canadian clinical trials have focused on validation of “poop pills” for other solid tumour malignancies [52,53], with an ongoing trial focusing on the efficacy for PDAC (NCT06393400). Alternatively, an immune response may be effectively mounted by personalized mRNA neoantigen vaccines as described by Rojas et al. [54]. Taken together, recent research initiatives point to increasingly individualized approaches for prognostication and treatment with a common goal of finally leveraging immunotherapy.

Discussions revealed an observation of low enrollment in clinical trials. This is owing to a false stigma that they are a “last ditch effort” and overburdened oncologists lacking the time to review potential trials based on individualized eligibility. Clinical trial participation offers patients treatment that is anticipated to be at least as effective as the standard of care based on previous phases of the trial and supporting research, with the benefits of more stringent monitoring. Eligibility is often maximized prior to receiving any other treatment, so it is imperative that clinical trials are discussed early following a patient’s diagnosis. Future advocacy efforts are encouraged to educate the general public on the benefits of clinical trials, so newly diagnosed patients are better equipped to propose this avenue of treatment upon diagnosis.

## 6. Advances in Neoadjuvant Therapy

Prior to the early 2010s, combined surgery and chemotherapy regimens focused on employing chemotherapy in the adjuvant setting. The CONKO-001 trial validated the use of adjuvant Gemcitabine [55]. PRODIGE24 demonstrated the disease-free and overall survival benefits of FOLFIRINOX over Gemcitabine [56]. And APACT and ESPAC4 improved upon Gemcitabine alone by combining with nab-Paclitaxel [57] and capecitabine [58], respectively. Current guidelines for patients with resectable disease suggest upfront surgery followed by FOLFIRINOX unless the patient exhibits lower performance status, in which case they should receive Gemcitabine-based adjuvant therapy [59]. However, recent research points to improved outcomes from a neoadjuvant approach, making potentially curative surgery accessible to more patients and improving surgical outcomes. Recent studies evaluating various neoadjuvant regimens were discussed. The benefits of neoadjuvant therapy compared to upfront surgery followed by adjuvant chemotherapy for resectable disease were presented based on findings from the PREOPANC and NORPACT trials. Results from PREOPANC demonstrated a tripling in the five-year survival frequency and a 1.4-month increase in median overall survival following a neoadjuvant course of Gemcitabine-based chemoradiotherapy compared to upfront surgery [60]. By contrast, NORPACT-1 pointed to improved overall survival following upfront surgery compared to neoadjuvant FOLFIRINOX; however, the study was limited by variable degrees of completion across groups and a relatively short regimen of neoadjuvant therapy (2 months) [61]. In 2022, the phase II NEONAX trial aimed to compare Gemcitabine nab-Paclitaxel in the neoadjuvant and adjuvant settings. Although the trial failed to meet its prespecified primary endpoint (18-month survival), the study demonstrated the benefits of neoadjuvant Gemcitabine-based chemotherapy for resectable disease where R0 resection rates improved by 31% [62]. The study also highlighted improved median overall survival in the neoadjuvant group, albeit the difference was confounded by greater rates of completion for neoadjuvant approaches (84.7%) vs. post-operative chemotherapy (25%). The SWOG1505 trial compared FOLFIRINOX and Gemcitabine nab-Paclitaxel in the neoadjuvant setting for resectable disease, with no statistically significant difference in overall or progression-free survival between the two therapies [63]. Ongoing trials (PREOPANC-3 and ALLIANCE) are working to evaluate the benefits of neoadjuvant FOLFIRINOX vs. upfront surgery for resectable disease.

While randomized phase III trials continue to make incremental advances on recommendations for optimized neoadjuvant approaches for resectable disease, decision making for borderline-resectable disease was described to be more subjective. Patel et al. define borderline-resectable disease based on a loss of the normal tissue plane between the tumour and surrounding blood vessels and/or venous involvement of the superior mesenteric vein (SMV), portal vein (PV), or SMV-PV confluence, where reconstruction appears possible with no evidence of metastases [64]. However, discussions revealed that the definition of borderline-resectable disease varies across centres, acknowledging the importance of biochemical factors and patient fitness. These discrepancies support a need for improved standardization in surgical treatment of PCMore recently, the phase III trial optimized progression-free survival using FOLFIRINOX in the neoadjuvant setting vs. Gemcitabine for locally advanced PC [65].

Neoadjuvant therapy remains a pillar of current research and clinical trials, but benefits for surgical outcomes, reduction of post-operative complications, and overall survival have been described. Current initiatives aim to further optimize neoadjuvant therapies, leveraging combination cytotoxic/immunotherapy regimens (DORA trial [66]) as well as the determination of genetic biomarkers indicative of optimal response to a neoadjuvant approach. Limitations such as lower access to targeted therapies (relying on a bulk sample of the resected tumour) and a delay of surgery were discussed. However, operating room availability is a common bottleneck, so future studies could investigate the frequency of neoadjuvant therapy effectively delaying surgery.

## 7. Regulatory Reform

PC is plagued with later diagnosis and higher mortality rates than most other cancers [1], necessitating highly effective treatment including first- and second-line options. The development of novel treatment options has lagged for PC compared to other cancers, owing in part to its resistance to immunotherapy, which leaves most patients reliant on FOLFIRINOX and/or Gemcitabine nab-Paclitaxel. However, the NAPOLI trial demonstrated the benefits of a new cytotoxic therapy, irinotecan liposome injection (Onivyde) for treatment of metastatic PC, and ongoing trials show promise for new immunotherapy and targeted therapy approaches. The impact of emerging new therapies can only be fully realized if they are accessible to patients. The pathway from Health Canada approval to patient access (with re-imbursement) was thoroughly discussed.

Canada is well-known for its universal healthcare system; however, there are inefficiencies within the pipeline to gaining access to drugs (recently reviewed by Gotfrit et al. [67]) that leave much to be desired [68].

Canada ranks 19th out of 20 OECD countries for the time patients wait to gain access to approved drugs. Health Canada approval took an average of 300 days in 2024, while re-imbursement took an average 598 days (over 2 years total elapsed time). Canada’s drug system consists of clinical trial review and regulatory review by Health Canada, a health technology assessment by Canada’s Drug Association, and price negotiation (considering patient and clinician input) by the pan-Canadian Pharmaceutical Alliance. This elapsed time for federal approval is followed by provincial reviews, resulting in the drug being variably reimbursed across Canada. Provinces are encouraged to adopt “fast track” programmes to help expedite their own reviews of new drugs. The current lengthy review processes were described to impede drug developers from even presenting their products to Health Canada.

The average Canadian cancer patient faces over $32,000 in cancer-related expenses including travel, accommodations, childcare, and lost income. With greater financial hurdles, patients are known to miss appointments, neglect to fill prescriptions, and compromise between receiving their treatment and fulfilling basic needs such as nutrition, which was noted in Section 4 to play a particularly critical role in the management of PC. Considering the challenges associated with any cancer diagnosis, equitable drug access should be a given in Canada. Future advocacy efforts are encouraged to elicit policy change, equitable access to genomic sequencing, and a comprehensive analysis of Canada’s drug budget against the cost of hospitalization.

## 8. Conclusions and Next Steps

Progress in the treatment and management of PC continues to be driven by the ongoing development of targeted therapies, immunotherapy (e.g., through vaccines, modulation of the tumour microbiome), nutrition/PERT support, and individualized consideration for surgical and neoadjuvant approaches. Variable practices across Canada necessitate the development of national standards/guidelines for the diagnosis/screening and treatment/management of PC followed by individualized provincial pathways guiding their execution. Given the pace of research and clinical trials, these standards will require revisions at defined intervals to maintain alignment with current best practices. Considering the progress in development of targeted therapies, early iterations of the national standards will surely reflect a need for equitable access to timely germline and somatic genetic sequencing/molecular profiling. Once accepted by healthcare providers and allied professionals, the published guidelines are anticipated to serve as a strong advocacy tool for improved drug access. Current evidence points to optimized PC treatment and management in a dedicated multidisciplinary centre, exemplified in Canada by the Baker Centre for Pancreatic Cancer (London, ON) and the Wallace McCain Centre for Pancreatic Cancer (Toronto, ON). Other provinces are encouraged to adopt similar models, leveraging the benefits of an integrated network of surgeons, oncologists, dietitians, and supportive care professionals.

Many themes from the NPC were heavily clinical; however, the importance of upstream foundational research can not be overstated. PC is notoriously challenging to treat owing to limited targetable mutations, dense stroma, and an immunosuppressive tumour microenvironment. Thus, research initiatives that aim to surmount these barriers including KRAS-driven pathology [69,70], probing the tumour microenvironment [71,72,73], and the role of specific ion channel dysregulation [74,75,76,77] are important topics to continue to explore. The continuation of foundational research holds a permanent place among the critical “next steps” towards improving outcomes for PC patients.

## Figures and Tables

**Table 1:** Germline and somatic mutations and associated recommended therapies.

Germline Mutation	Somatic Mutation	Recommended Therapy
BRCA1/2, PALB2		Platinum-based, maintenance PARP inhibitor
	KRAS G12C	KRAS G12C inhibitors
	RAS G12	Daroxanrasib
	BRAF V600E	BRAF/MEK inhibitor
	High microsatellite instability/misMatch repair deficiency	Pembrolizumab
	NTRK	TRK inhibitor
	HER2	HER2-targeted therapy
	High tumour mutation burden	Potential immunotherapy

## Data Availability

No new data were created or analyzed in this study. Data sharing is not applicable to this article.

## Supplementary Materials

The following supporting information can be downloaded at https://www.mdpi.com/article/10.3390/curroncol33100629/s1, File S1: Summary of National Pancreas Conference 2025 Needs Assessment; File S2: National Pancreas Conference 2025 Conference Programme.

## Abbreviations

The following abbreviations are used in this manuscript:

CAGS	Canadian Association of General Surgeons
FMT	Fecal microbiota transplantation
mRNA	Messenger ribonucleic acid
NPC	National Pancreas Conference
OECD	Organisation for Economic Co-operation and Development
OS	Overall survival
PC	Pancreatic cancer
PDAC	Pancreatic ductal adenocarcinoma
PEI	Pancreatic exocrine insuffiency
PERT	Pancreatic enzyme replacement therapy
PV	Portal vein
SMV	Superior mesenteric vein
