# Supplementary material for: Canadian National Pancreas Conference 2025: Recent Advances in Patient-Centred Management of Pancreatic Cancer
Source: Curr Oncol. 2026 Oct 19;33(10):629. doi: 10.3390/curroncol33100629 (PMC13703088; doi:10.3390/curroncol33100629)
Supplement: Supplementary file 1 [file curroncol-33-00629-s001.zip › File S1 Summary of National Pancreas Conference 2025 Needs Assessment.pdf]

Summary of
National Pancreas Conference 2025
Needs Assessment

Respondents

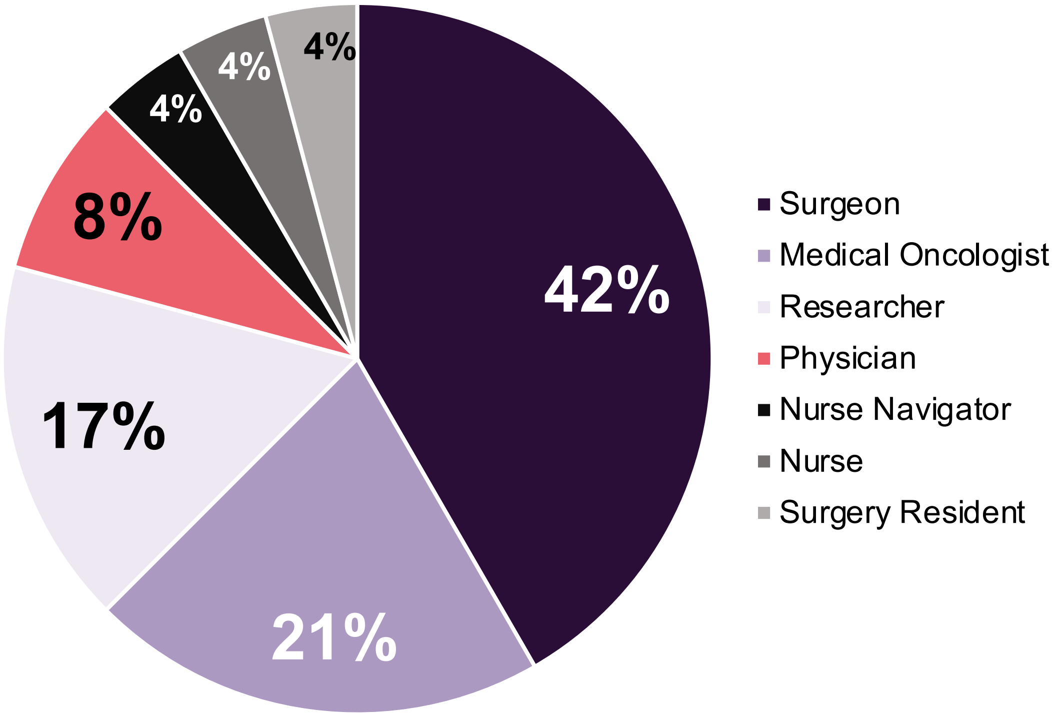

The Needs Assessment survey was completed by 25 respondents. 76% were surgeons, medical oncologists, or researchers. 2 general physicians, 1 nurse navigator, and 1 surgery resident were also represented.

84% of respondents were working in an urban area, while 16% were in a rural setting.

Self-reported familiarity with best practices

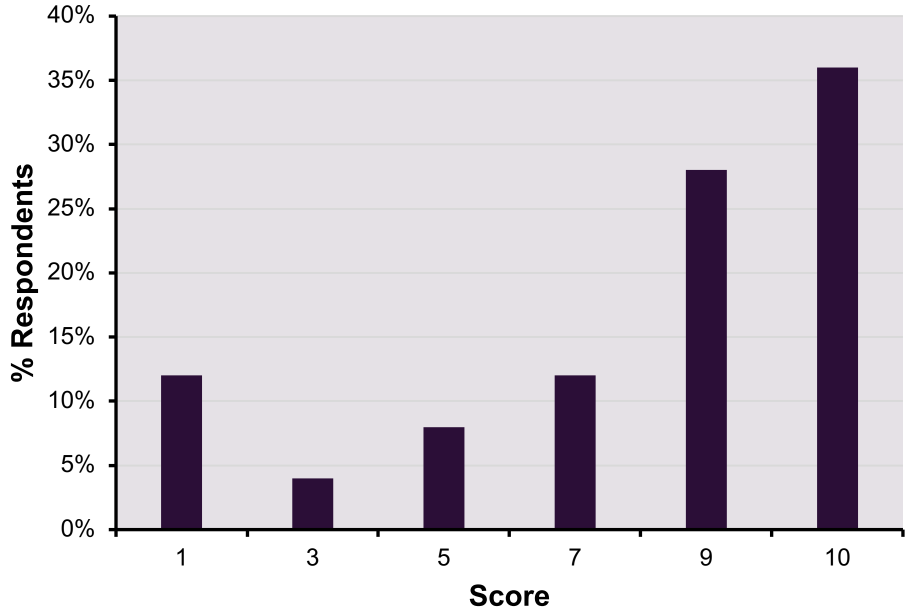

Respondents were asked to rank their familiarity with the Western Canadian Gastrointestinal Cancer Consensus Conference and American Society of Clinical Oncology best practices (10 – expert, 1– unfamiliar). 35% indicated they are highly familiar with current best practices while 65% of respondents show room for better education on these recommendations towards reaching a national standard.

Referrals

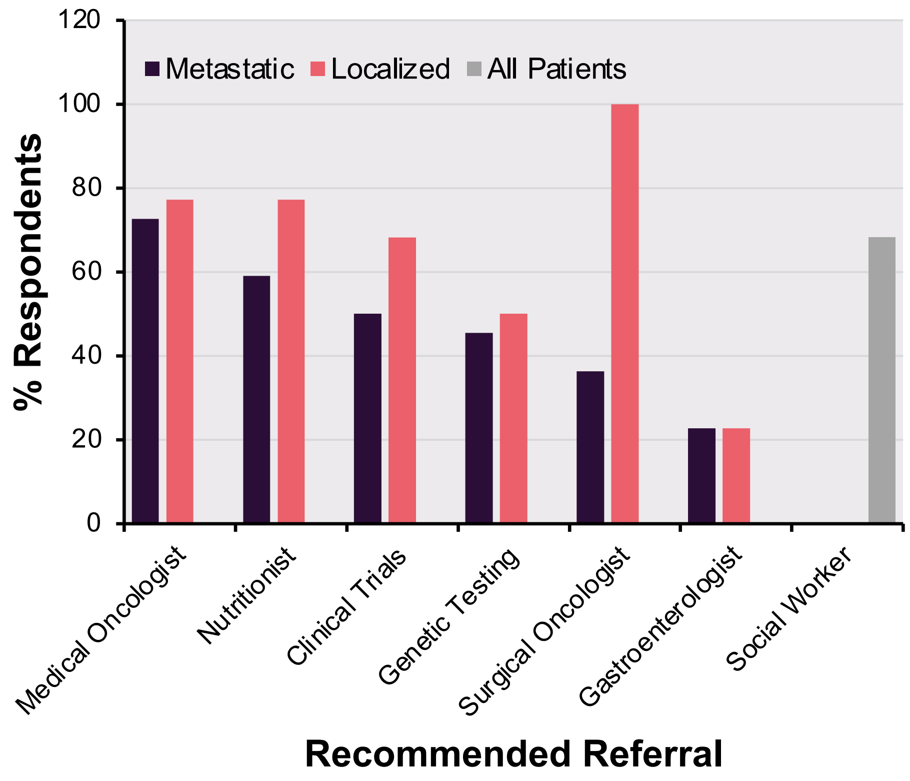

The recommended referrals point to enhanced support for patients with localized disease vs. those with metastatic disease. This contradicts the current best practices which suggest that all patients should be referred to a medical oncologist, an HPB surgeon, clinical trials, and nutrition.

The gaps in recommended referrals demonstrate a need for further education on the supports that pancreatic cancer patients need. Furthermore, referrals should not be reduced based on an assumption the patient won't be seen in a timely manner. The demand needs to be there in order to advocate for improved resources.

When should genetic testing be referred?

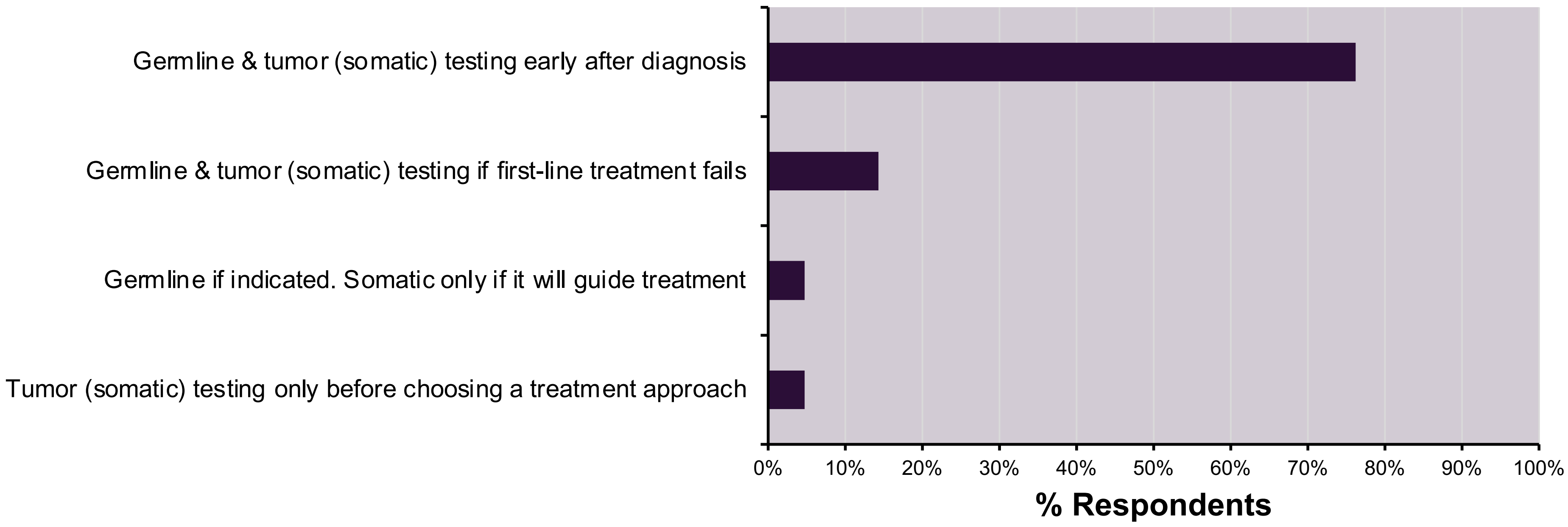

A promising 76% of respondents recommended referring all patients for germline and somatic genetic testing soon after diagnosis. 14% recommended looking at genetic testing after exhausting first-line treatment. It is apparent that the importance of genetic testing early in a patient's journey is almost unanimous, but these results support an interest in education on the latest advances in targetable mutations.

When should palliative care be referred?

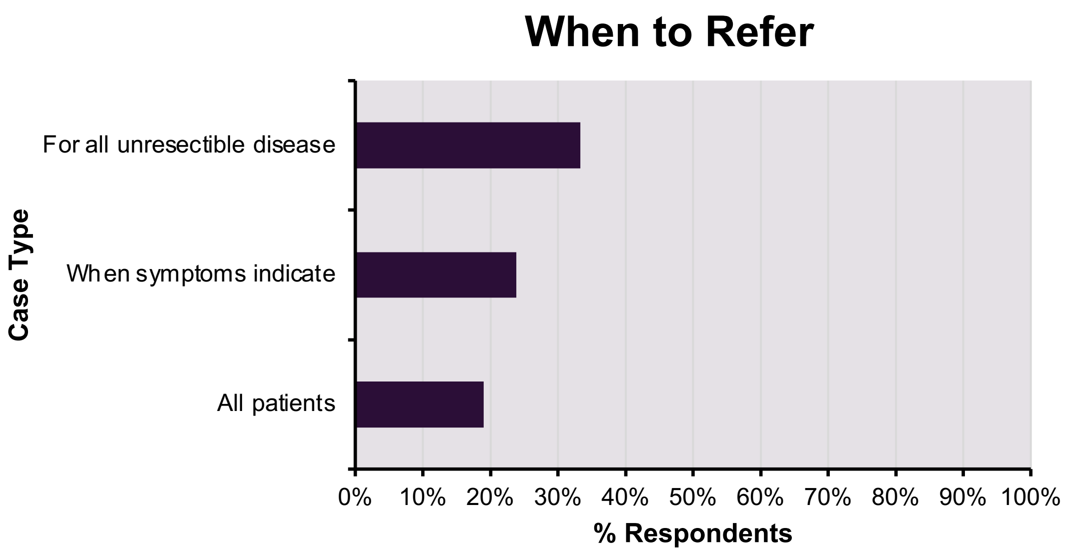

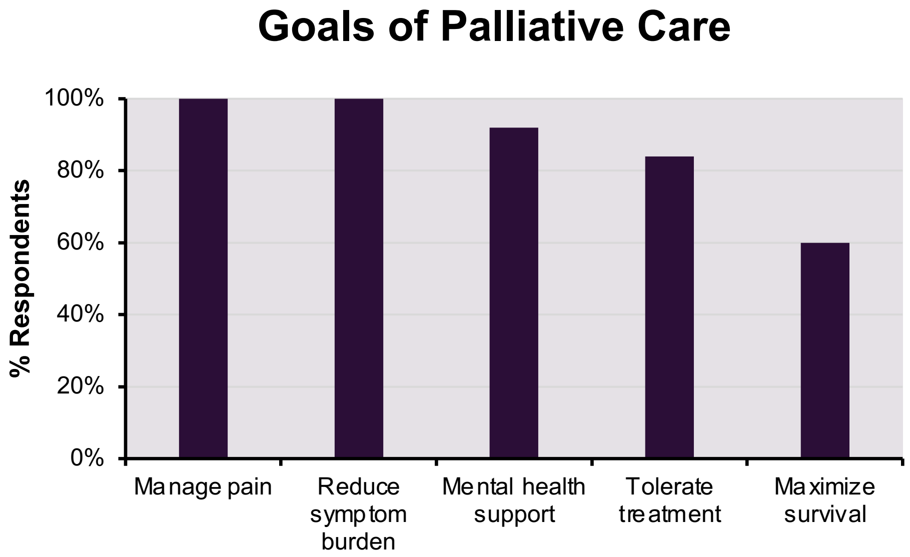

The most common recommendation was to refer all metastatic patients for palliative care (33% of respondents). Another 19% recommend referring all PC patients for palliative care. The remaining 24% suggest that palliative care should be referred in response to unmanaged symptoms. The lack of agreement across respondents demonstrates the need for clarification on the best practices as well as the benefits of palliative/supportive care throughout a PC journey.

In terms of the perceived goals of palliative care, there is a unanimous understanding the palliative care can manage pain and reduce symptom burden, however, other potential goals are underrepresented with maximized survival at 60%, treatment tolerance at 84%, and mental health support at 92%. These gaps demonstrate a need for further clarification on the diverse and personalized goals/benefits of a palliative approach.

Systemic Barriers

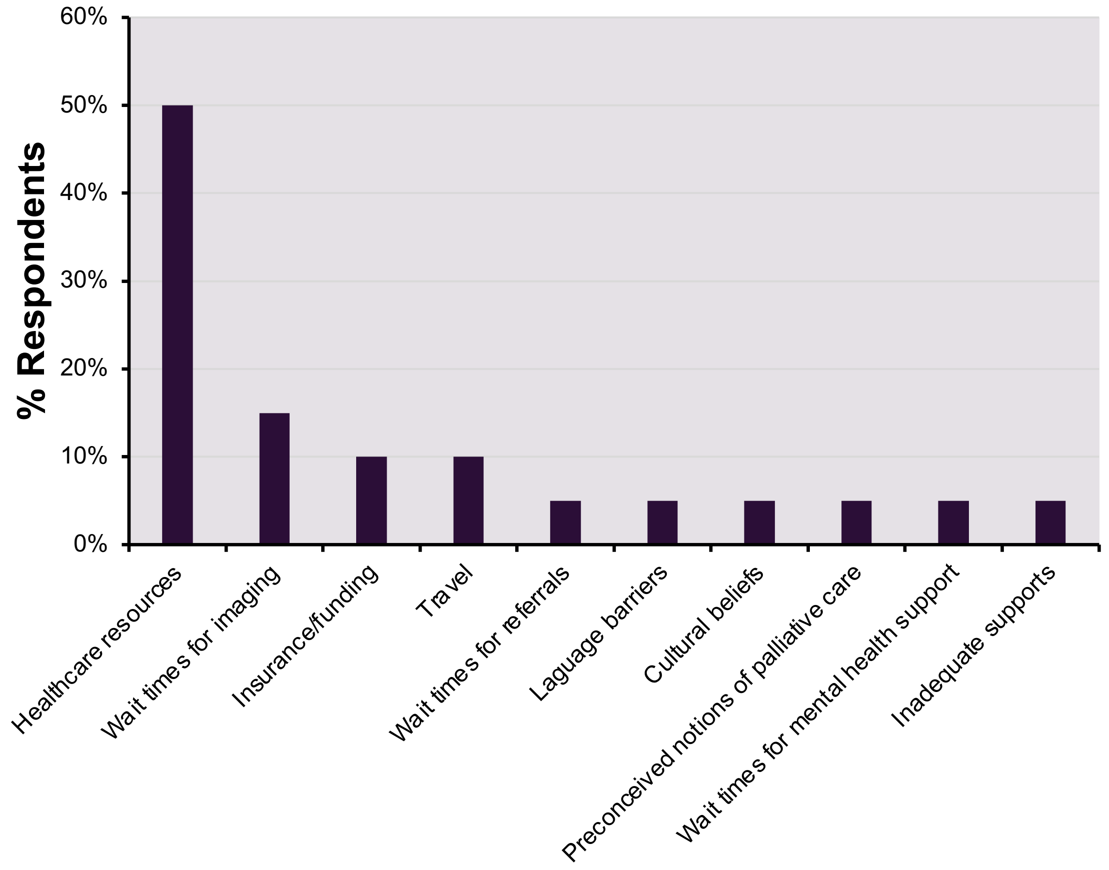

75% of respondents indicated limitations in healthcare resources which results in longer wait times for referrals, imaging, and other supports.

Other barriers include the financial strain on patients (travel to treatment and insurance coverage).

These findings demonstrate a need for a discussion on how to optimize the limited resources available and how advocacy organizations can push for improvements.

Ranking interest

1. ***Neoadjuvant therapy***
2. ***Genomic & molecular profiling***
3. ***Advances in immunotherapy***
4. ***Advances in surgical resection***
5. ***Clinical trials***
6. ***Patient-centered palliative care***
7. ***Nutrition & PERT***
8. ***Screening & detection***
9. ***Mental health support***

Respondents were asked to rank their interest in the listed topics (1 – most interested, 9 – least interested). The proposed agenda was sure to cover the top indicated topics. However, the low ranking of nutrition & PERT points to some underappreciation of its critical role and the challenges associated with optimizing dosage. The complementary literature review was also taken into consideration when discerning the most relevant and necessitated topics.
