# Supplementary material for: Canadian National Pancreas Conference 2025: Recent Advances in Patient-Centred Management of Pancreatic Cancer
Source: Curr Oncol. 2026 Oct 19;33(10):629. doi: 10.3390/curroncol33100629 (PMC13703088; doi:10.3390/curroncol33100629)
Supplement: Supplementary file 1 [file curroncol-33-00629-s001.zip › File S2 National Pancreas Conference 2025 Conference Programme.pdf]

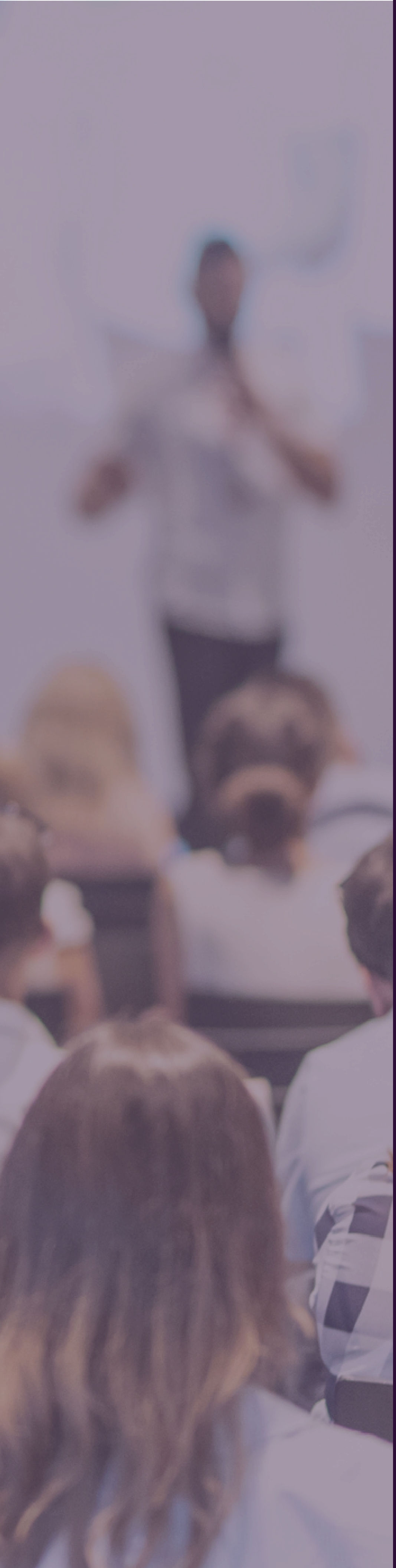

NATIONAL PANCREAS CONFERENCE 2025

NATIONAL PANCREAS CONFERENCE
NOVEMBER 21-23

Presented by:

Craigs Cause Pancreatic Cancer Society
London Health Networks

Craig's Cause
Pancreatic Cancer Society

London Health Sciences Centre
Baker Centre for Pancreatic Cancer

Craig's Cause Pancreatic Cancer Society and The Canadian Association of General Surgeons is pleased to welcome you to the 2025 National Pancreas Conference.

Craig's Cause
Pancreatic Cancer Society

CANADIAN ASSOCIATION OF
General Surgeons

London Health Sciences Centre
Baker Centre for Pancreatic Cancer

London, Ontario

The National Pancreas Conference 2025 is offered as a hybrid conference, both, in-person and virtual.

TABLE OF CONTENTS - NATIONAL PANCREAS CONFERENCE 2025

**WELCOME**	2
**GENERAL INFORMATION**	3
**PROGRAM INFORMATION**	4
**RESEARCH GRANTS**	5
**AGENDA**	6
**SCIENTIFIC PLANNING COMMITTEE**	7
**PRESENTATIONS**	8
• Patient Perspectives: Key Findings from Canada's Pancreatic Cancer Survey	9
• Driving Change: Advocacy for Regulatory Reform and Funding in Pancreatic Cancer	10
• Building Consensus: Shaping Canada's Pancreatic Cancer Care Pathways	12
• Precision Power: Genomic Testing and Targeted Therapies in Pancreatic Cancer	14
• Unlocking New Horizons: Pancreatic Cancer Clinical Trials and Advances Across Canada	16
• Neoadjuvant Therapy: Transforming the Surgical Landscape of Pancreatic Cancer	18
• Proven Models, National Impact: Expanding Patient-Centered Pancreatic Cancer Care	20
• Innovations Unveiled: Poster Presentations by Rising Scientists	22
• Not Optional: Making Nutrition and PERT Standard in Treatment Pathways	23
• From Protocol to Progress: The Therapies Transforming Survival and Quality of Life	24
• Next Steps: Driving Impact and Innovation for Pancreatic Cancer Care	25
**INDUSTRY SPONSORS & PARTNERS**	27

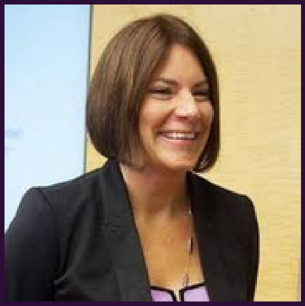

Dear Participants,
It is with great pleasure that I welcome you to Canada's only disease-specific, accredited **National Pancreas Conference 2025**. The 2023 National Pancreas Conference created an opportunity for healthcare professionals and advocates from across Canada to come together and address the many challenges facing patients, caregivers, advocates, and healthcare providers. Not only was a conference review paper published, but the recommendations generated in 2023 guided meaningful national action over the past two years.

Following the conference, we established dedicated steering committees to advance these recommendations. Together, we have:

- Completed a national **Myth Buster Social Media Campaign** on clinical trials, reaching and educating thousands of Canadians.
- Developed **draft national pathways for Diagnosis, Treatment, and Management**, and PERT providing a more consistent and equitable approach to patient care across provinces.

We are excited to share these drafts with you this weekend. Your expert input is essential as we work toward finalizing national pathways that reflect best practices, increase survival rates, and improve the quality of life for all Canadians diagnosed with pancreatic cancer—regardless of where they live.

We are also thrilled to welcome an exceptional group of speakers and leaders who will contribute their knowledge, experience, and passion to this year's discussions.

Thank you for being part of this vital work. We look forward to a productive, collaborative, and inspiring 2025 National Pancreas Conference.

Stefanie Condon-Oldreive
Founder
Craig's Cause Pancreatic Cancer Society

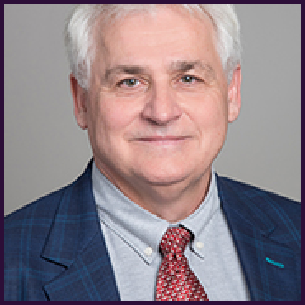

On behalf of the Baker Center for Pancreatic Cancer, Verspeeten Family Cancer Center, London Health Science Center and Westen University I would like to welcome the 2025 National Pancreatic Conference to London. This conference brings together a multidisplinary team to discuss the care of pancreatic cancer patients and research into the disease.

We acknowledge the great dedication that Craig Cause Pancreatic Cancer Society does to organize and run this very important meeting. We are looking forward to an exciting meeting.

Dr. Ken Leslie, MD, FRCSC
HPB Surgeon, Chair of Scientific Planning Committee
London Health Sciences Centre

Virtual Information -Online Protocol

- Remember to post your name on your Zoom account to facilitate our attendance records, so participants can claim Section 1 MOC credits
- Keep your camera on, so our presenters can see their audience.
- Each presentation will have an interaction component for asking questions and collaborating.
- If you have a question, during the presentation, type it into our chat. Our moderators will ensure your question will be answered.
- For Technology Support, a phone number and email will be provided prior to the conference.
- Participants are encouraged to post and engage in discussions about presentations and collaborations!

NPC 2025 Venue

Best Western Plus Lamplighter Inn & Conference Centre
591 Wellington Rd
London, ON N6C 4R3

Badges

NPC name badges are non transferable and to be worn and visible at all times by all delegates. They are required to access sessions and social functions.

Social Media #NPC2025

@CraigsCauseCan

www.linkedin.com/company/craig-s-cause-pancreatic-cancer-society

Learning Objectives for the National Pancreas Conference

- Summarize national statistics on patient-centered care gaps impacting pancreatic cancer management and supportive care strategies
- Discuss recent evidence on regulatory frameworks and advocacy approaches towards influencing timely access to novel pancreatic cancer therapies in Canadian healthcare
- Summarize standardized diagnostic, treatment, and management pathways incorporating precision medicine, genomic profiling, and targeted therapies
- Integrate emerging research findings and multidisciplinary clinic models to improve pancreatic cancer treatment and patient care delivery

Accreditation Statement (Pending accreditation approval)

This event is an Accredited Group Learning Activity (Section 1) as defined by the Maintenance of Certification Program of the Royal College of Physicians and Surgeons of Canada and approved by the Canadian Association of General Surgeons. You may earn a maximum of 14.75 hours.
This event was co-developed with the Canadian Association of General Surgeons and was planned to achieve scientific integrity, objectivity and balance.

Certificate of Attendance

All delegates attending the National Pancreas Conference will receive a Certificate of Attendance. Following the conference an email will be sent with the information for you to document your credits.

Session Evaluation

We value your feedback! At the conclusion of each session participants will be asked to complete a short evaluation. At the end of the conference, once again participants will be asked to complete an evaluation on the event as a whole.

Disclosure of Conflict of Interest

The National Pancreas Conference has a formal policy regarding the need for authors and presenters to inform the National Pancreas Conference of any conflict of interest (COI). A COI includes, but is not limited to, employment, ownership of stock, membership on a standing advisory council or committee, being on the board of directors, publicly associated with a company or its products. Other potential areas of real or perceived COI could include receiving honoraria, consulting fees, or grants.

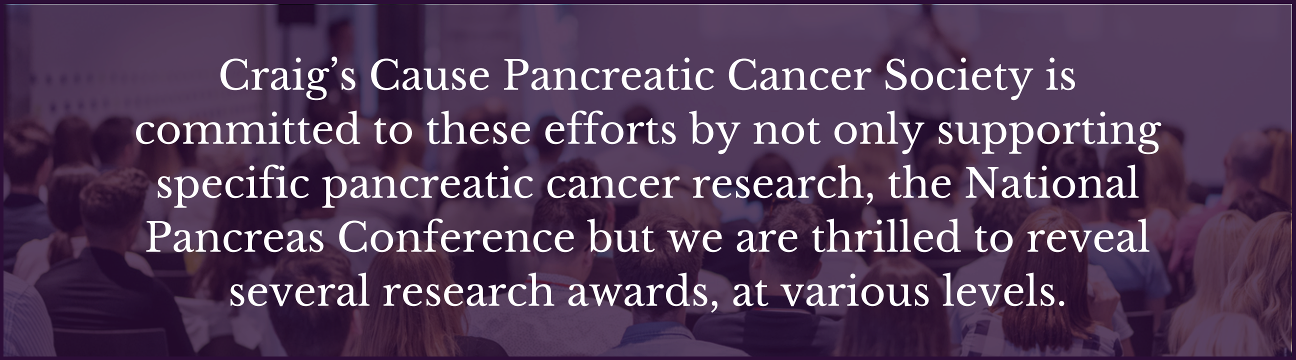

Pancreatic cancer is a multifaceted disease that demands treatment and management from a variety of sectors in the healthcare field, with additional strong networks between healthcare providers and foundational researchers. Many of the unique challenges associated with pancreatic cancer precipitate from late-stage diagnosis, molecular and cellular heterogeneity, and several morbidities that largely derive from malnutrition. As reviewed by Grossberg in 2020, a multimodal approach that integrates improved screening, targeted/precision medicine approaches to treatment (including in the palliative setting), and holistic management of symptoms has proven to increase metastasis-free survival and quality of life[1].

Advancements in treatment options for pancreatic cancer have not kept pace with those for other cancers[2], but efforts continue to adapt, develop and combine approaches to effect change in the difficult-to-treat cancer.

We encourage not only multi-disciplinary approaches and teams but also multi-disciplinary approaches that combine talent from more than one province!

1. Doleh, Y., Lal, L. S., Blauer-Petersen, C., Antico, G. & Pishvaian, M. Treatment patterns and outcomes in pancreatic cancer: Retrospective claims analysis. Cancer Med. 9, 3463–3476 (2020).
2. Grossberg, A. et al. Multidisciplinary standards of care and recent progress in pancreatic ductal adenocarcinoma. CA Cancer J Clin. 70, 375-403 (2020).

Winners will be awarded during conference

Poster Board Award
Award Amount: $750.00
Number of Awards: 2

The intent of the poster presentations is to encourage collaborative and interdisciplinary approaches that improve patient survival rates and quality of life.

Junior Research Grant
Award Amount: $7,500.00
Number of Awards: 2

The intent of the Junior Researcher Grant is to create opportunities that encourage collaborative and interdisciplinary approaches in the field of pancreatic cancer research. Applicants are encouraged to look at projects that will increase survival rates and improve quality of life.

Faculty Research Grant
Award Amount: $50,000.00
Number of Awards: 1

The intent of a Senior Research Grant opportunity is to encourage collaborative and interdisciplinary approaches that improve patient survival rates and quality of life are key to this research grant opportunity.

AGENDA

21 Friday

6:30–8:00 PM	Patient Perspectives: Key Findings from Canada's Pancreatic Cancer Survey. Canada's first national survey: top insights from patients and caregivers.

22 Saturday

8:30–10:00 AM	Driving Change: Advocacy for Regulatory Reform & Funding in Pancreatic Cancer
10:15–11:15 AM	Building Consensus: Shaping Canada's Pancreatic Cancer Care Pathways
11:30 AM–1:00 PM	Precision Power: Genomic Testing and Targeted Therapies in Pancreatic Cancer
1:00–2:00 PM	Lunch (provided)
2:00–3:00 PM	Unlocking New Horizons: Pancreatic Cancer Clinical Trials and Advances Across Canada
3:00–4:00 PM	Neoadjuvant Therapy: Transforming the Surgical Landscape of Pancreatic Cancer
4:00–5:30 PM	Proven Models, National Impact: Expanding Patient-Centered Pancreatic Cancer Care
5:00–7:00 PM	Poster Presentations. Residents and trainees share cutting-edge research.
6:00–8:00 PM	Dinner (provided)

23 Sunday

8:30–10:00 AM	Not Optional: Making Nutrition and PERT Standard in Treatment Pathways
10:15–11:30 AM	From Protocol to Progress: Therapies That Move the Needle. Current surgical/systemic standards with the strongest evidence.
11:30 AM–12:00 PM	Next Steps: Driving Impact & Innovation. Closing panel: priorities for research, advocacy, and care delivery.

Craig's Cause Pancreatic Cancer Society is incredibly grateful to the volunteer members of the National Pancreas Conference 2025 Scientific Planning Committee. It is their guidance and expertise that has made this third NPC event possible.

Chair

Dr. Ken Leslie, MD, FRCSC
Hepato-Pancreato-Biliary Surgeon

Committee Members

Stefanie Condon-Oldreive, Founder/Director
Craig’s Cause Pancreatic Cancer Society

Jessica Nickerson, PhD, Healthcare Education Rep.,
Craig's Cause Pancreatic Cancer Society

Ken Leslie, MD, FRCSC, Ontario

Ravi Ramjeesingh, MD, PhD, FRCPC, Nova Scotia

Lee-Hwa Tai, PhD, Québec

George Zogopoulos, MD, PhD, FRCSC, FACS, Québec

Joan Miguel Romero, MD Candidate, Québec

Somtochukwu Ibezi, MD, Saskatchewan

Rodney Ouellette, MD, PhD, New Brunswick

Winson Cheung, MD, MPH, FRCPC, Alberta

Stephen Quigley, MD, Newfoundland

Jennifer Campbell, Ontario

PRESENTATIONS

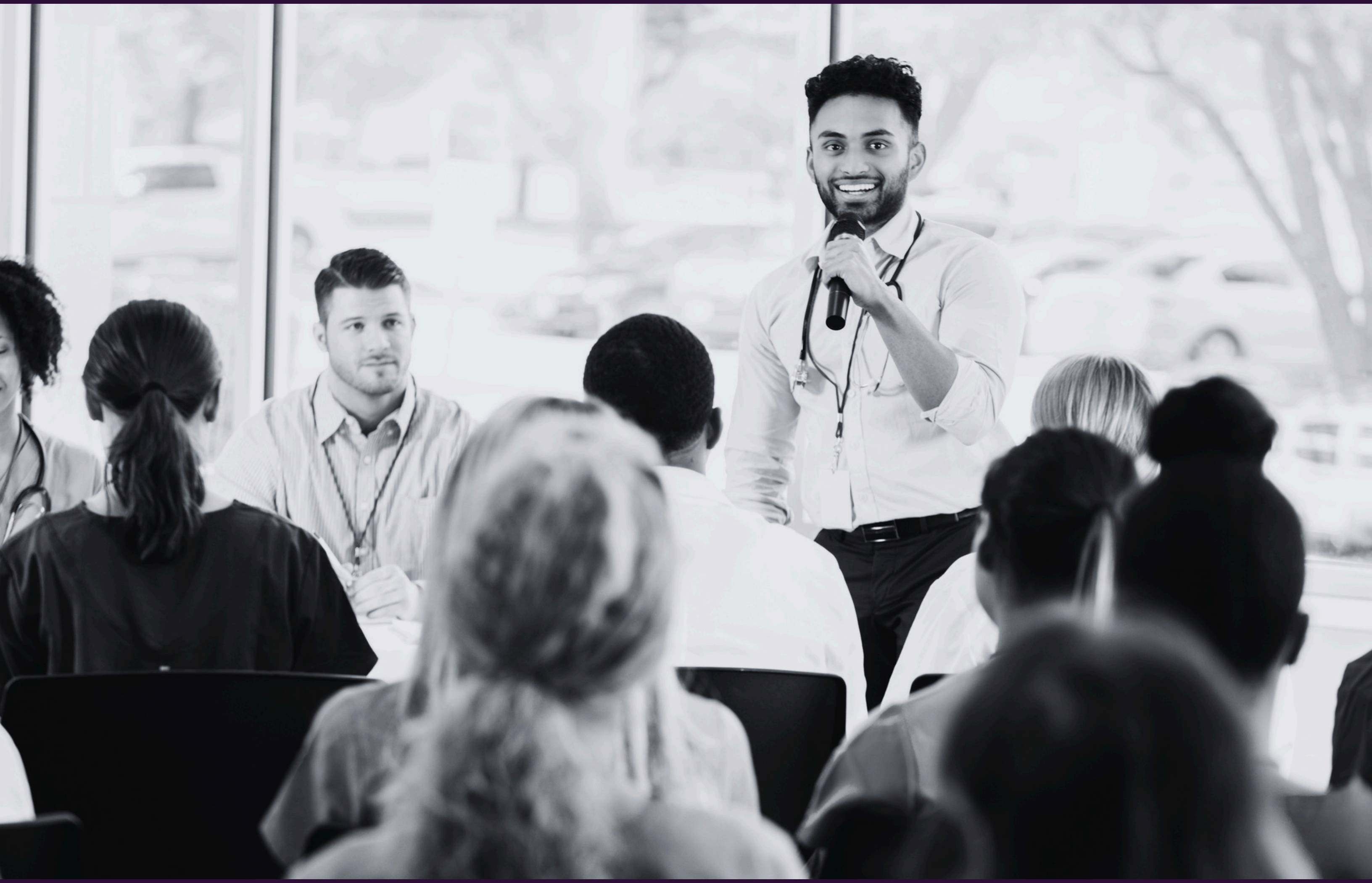

NATIONAL PANCREAS CONFERENCE
NOVEMBER 21-23

Patient Perspectives: Key Findings from Canada’s Pancreatic Cancer Survey

Presented by Craig’s Cause Pancreatic Cancer Society

PRESENTATION SUMMARY:
Discover insights from Canada’s first national survey focused on the pancreatic cancer experience. This groundbreaking data highlights patient and caregiver challenges, gaps in care, and opportunities for support across the disease journey—informing advocacy, research, and healthcare priorities nationwide.

LEARNING OBJECTIVES:
By the end of this session, learners will be able to:
- Understand key challenges faced by pancreatic cancer patients and caregivers across the care continuum, as revealed by Canada's first national survey.
- Identify gaps in care and support that can inform improvements in healthcare delivery, advocacy efforts, and research priorities.
- Discuss how patient-reported experiences can shape national strategies, policy development, and equitable access to care.

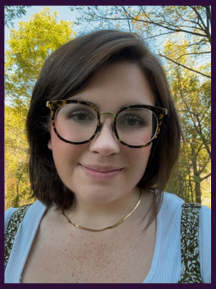

Erin Ranger
Craig’s Cause Pancreatic Cancer Society

Erin Ranger joined Craig's Cause Pancreatic Cancer Society in the fall of 2022 after seven years of nursing experience across several areas, including the operating room. No matter the setting, she has always been drawn to supporting people through stressful, confusing, and vulnerable moments. As Patient Support Manager, Erin hopes to be a steady and caring presence for those affected by pancreatic cancer. She focuses on meeting people where they are and offering education, connection, and compassionate support so patients and families feel understood and less alone in what they’re facing.

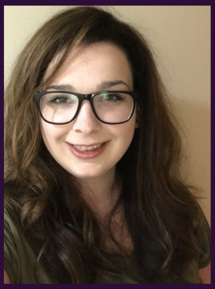

Jessica Nickerson
Craig’s Cause Pancreatic Cancer Society

Jessica Nickerson is a Healthcare Education Representative at Craig’s Cause Pancreatic Cancer Society and an analytical scientist by trade. She completed her PhD in Analytcal Chemistry at Dalhousie University with a focus on proteomics method development in 2022. She has since been working as a Scientist at Allumiqs and has been involved in the Healthcare Education initiatives at Craig’s Cause Pancreatic Cancer Society since 2020.

Driving Change: Advocacy for Regulatory Reform and Funding in Pancreatic Cancer

Jim Lambley
Canadian Drug Agency

Louise Binder
All.Can Canada

PRESENTATION SUMMARY:
Collaboration between the healthcare sector and advocacy groups is vital to ensure timely access to new treatments. By aligning efforts, they can influence regulatory reviews and provincial funding decisions, helping bridge delays between drug approval and reimbursement—ultimately improving outcomes for patients across Canada.

LEARNING OBJECTIVES:
By the end of this session, learners will be able to:

- Examine the regulatory and reimbursement processes for new cancer treatments in Canada and the roles of key decision makers.
- Identify strategies for effective collaboration between advocacy organizations, clinicians, and policymakers to support equitable drug access.
- Understand how coordinated advocacy efforts can reduce delays between drug approval and public funding to improve patient outcomes.

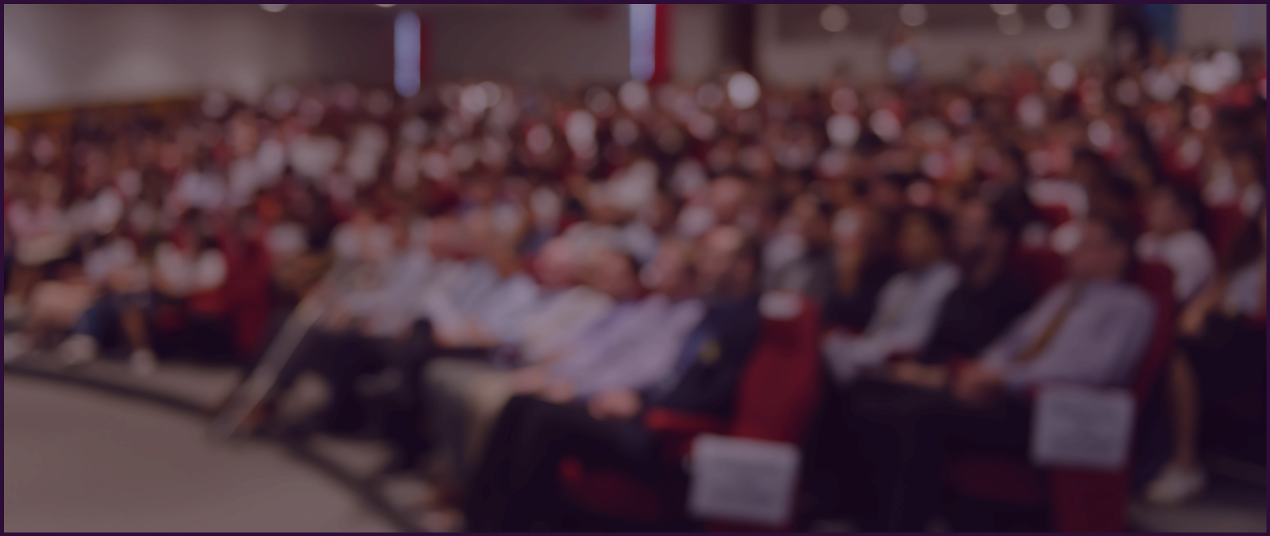

Driving Change: Advocacy for Regulatory Reform and Funding in Pancreatic Cancer

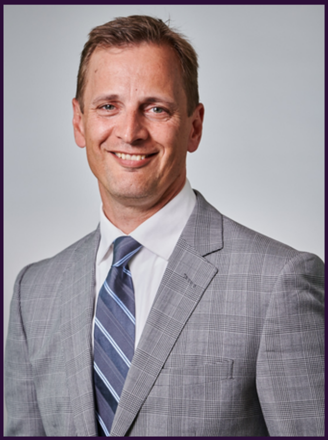

Jim Lambley
Canadian Drug Agency

Jim Lambley is the Manager of Patient, Clinician, and Industry Engagement at Canada's Drug Agency (CDA-AMC). Before joining the pan-Canadian Health Organization, Jim worked across areas of Engagement, Quality Improvement, Strategy and Risk. At the University of Ottawa Jim teaches in the Master of Health Administration and Executive (MHA) courses. A Certified Health Executive with the College of Canadian Health Leaders, he earned his Master of Health Administration (MHA) from the University of Ottawa, Telfer School of Management. In his role, Jim leads an amazing team of Engagement Officers that bring the voice of interested parties to the many diverse projects of CDA-AMC. Jim's career has spanned many areas across healthcare and business in the private and public sectors, connecting the dots from engagement, strategy, and tactics.

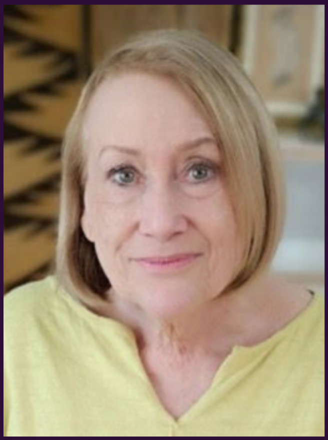

Louise Binder
All.Can Canada

Louise Binder is a lawyer and health policy advocate, developing policy and systemic treatment access for patients. She worked in HIV since 1994, co-founding the Canadian Treatment Action Council, ensuring access to treatments for patients. In 2017, Louise became Health Policy Consultant for Save Your Skin Foundation. She has received an Honorary Doctorate of Laws, the Order of Ontario, and two Queen Elizabeth II medals.

Building Consensus: Shaping Canada’s Pancreatic Cancer Care Pathways

Dr. Breffni Hannon
Princess Margaret Cancer Centre

Leah Stephenson
All.Can Canada

Jaymee Maaghop
Gastrointestinal (GI) Society

PRESENTATION SUMMARY:

Born from a key recommendation at the 2023 National Pancreas Conference, this draft framework outlines standardized steps in diagnosis, treatment, and management of pancreatic cancer. Join the discussion to provide input before final submission and help shape a unified approach to care across Canada.

LEARNING OBJECTIVES:

By the end of this session, learners will be able to:

- Review the proposed national pathways for pancreatic cancer diagnosis, treatment, and management developed from the 2023 conference recommendation.
- Identify gaps, strengths, and areas for refinement within the draft framework based on clinical experience and patient needs.
- Contribute to shaping a unified national approach that promotes consistency, equity, and improved outcomes in pancreatic cancer care across Canada.

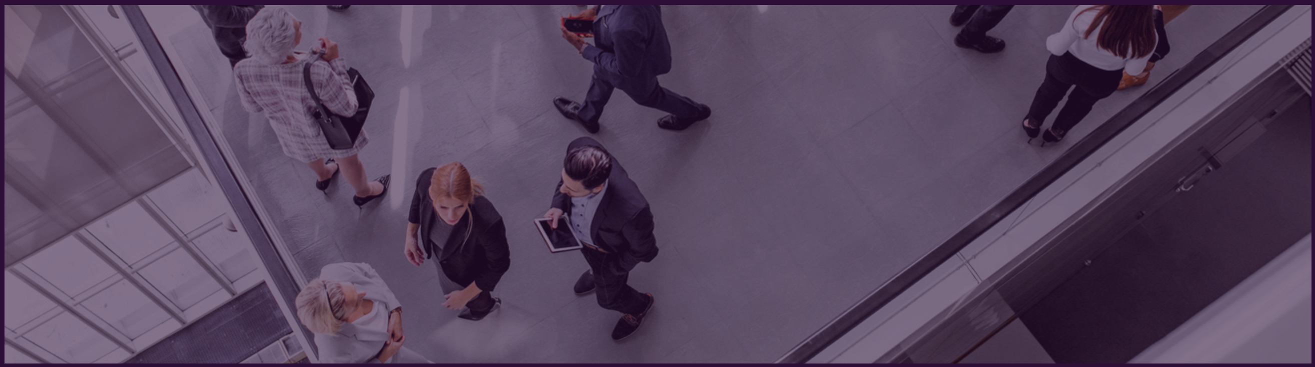

Building Consensus: Shaping Canada's Pancreatic Cancer Care Pathways

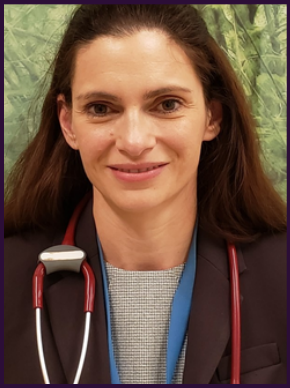

Dr. Breffni Hannon, Princess Margaret Cancer Centre

Dr. Breffni Hannon leads the palliative care program at the Princess Margaret Cancer Centre in Toronto. Originally from Ireland, she moved to Canada for a 2-year clinical-research fellowship in palliative care in 2011 and never left! She is a clinician-investigator whose research interests include developing models of early palliative care for patients with advanced cancer; exploring the needs of caregivers; and promoting advance care planning.

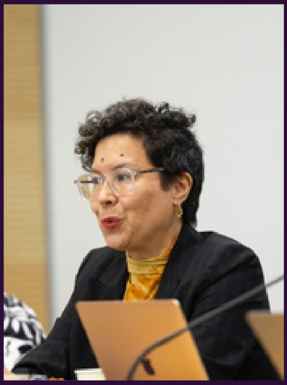

Leah Stephenson, All.Can Canada

Motivated by her experiences as a caregiver for her late mother, Leah Marie Migriño Stephenson has cultivated her consulting expertise to improve how we address complex systems change – as leaders, organisations, and collaboratives. She is the Strategic Lead of All.Can Canada, a patient-led, multi-stakeholder network focused on improving earlier, equitable cancer diagnoses in Canada. For almost twenty years, Leah has been working to improve Canadian healthcare systems, especially structurally underserved communities. Nationally, she has supported advocacy and improvements in treatment access and continues to advocate for pan-Canadian, interoperable, equity-informed, and community-governed health information. She has been the backbone support and pen behind the Declaration of Personal Health Data Rights in Canada endorsed by over 30 patient groups and Peel's Diversity and Inclusion Charter endorsed by over a 100 institutions, both of which continue to be brought to life through advocacy, education, and knowledge mobilization.

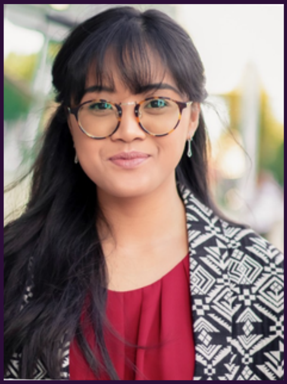

Jaymee Maaghop, Gastrointestinal Society

Jaymee Maaghop is the Health Policy and Outreach Manager for the Gastrointestinal Society. Connecting with patients and caregivers, she gathers their experiences with digestive and liver conditions as well as access to care and treatments. She actively writes patient input submissions to health technology assessment reviews, and works closely on several health policy files, including federal pricing regulations, medication supply and shortages, national pharmacare, standards of care, and more. Jaymee graduated from Carleton University with a Bachelor of Arts Honours in Law. She has spent the past eight years representing patient communities from various therapeutic areas, focusing on policy, advocacy, medical writing, and communications.

Precision Power: Genomic Testing and Targeted Therapies in Pancreatic Cancer

Dr. George Zogopoulos
McGill University Health Centre

Dr. Rodney Ouellette
Atlantic Cancer Research Institute

Dr. Orçun Haçariz
McGill University Health Centre

PRESENTATION SUMMARY:

Explore where Canada stands with research-based genomic testing and targeted therapies for pancreatic cancer. This session highlights the latest evidence demonstrating how precision medicine improves patient outcomes by tailoring treatments to individual tumour profiles, advancing more effective, personalized care through cutting-edge scientific research.

LEARNING OBJECTIVES:

By the end of this session, learners will be able to:

- **Understand the current landscape** of genomic testing and targeted therapy availability for pancreatic cancer patients in Canada.
- Evaluate the latest research evidence supporting precision medicine approaches and their impact on patient outcomes.
- **Identify challenges and opportunities** for integrating molecular profiling and liquid biopsy monitoring into standard pancreatic cancer care to improve personalized treatment.

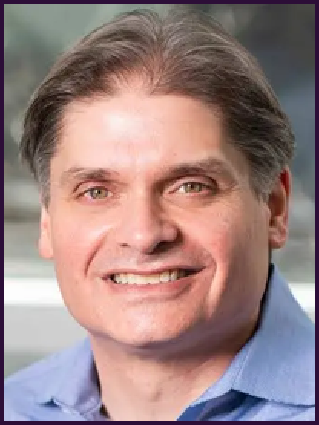

Dr. George Zogopoulos
McGill University Health Centre, QC

Dr. Zogopoulos is a surgeon-scientist with a translational research program on the genetics and oncogenomics of pancreaticobiliary cancers. His clinical practice focuses on the surgical treatment of pancreatic, biliary, and hepatic malignancies as well as abdominal organ transplantation with an interest in transplant oncology. He has been a junior and senior scholar of the Fonds de recherche du Québec and recipient of the Michal & Renata Hornstein Career Award for Surgical Excellence. Dr. Zogopoulos' leadership roles include co-Chair of the Pancreatic Disease Oriented Group of the Canadian Cancer Trials Group, Director of the Cancer Research Society, Quebec Regional Representative on the Steering Committee of the Marathon of Hope Cancer Centres Network, MUHC Institutional Lead for the Quebec Cancer Consortium and Marathon of Hope Cancer Centres Network, and Executive Member of the Pancreatic Cancer Early Detection (PRECEDE) Consortium.

Precision Power: Genomic Testing and Targeted Therapies in Pancreatic Cancer

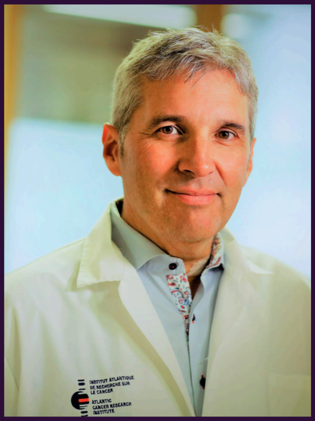

Dr. Rodney Ouellette
Atlantic Cancer Research Centre, NB

Dr. Rodney Ouellette is Founder and Senior Scientist of the Atlantic Cancer Research Institute (ACRI) in Moncton. He holds the R.R. Leger Chair in Precision Cancer Research. He also serves as the Medical Director of the Molecular Genetics and Sequencing Laboratory at the Dr. Georges-L.-Dumont University Hospital and is Adjunct Professor of Biochemistry at Université de Moncton. Dr. Ouellette received his BSc and MSc in Biochemistry from Université de Moncton, and his MD and PhD in Molecular and Cellular Biology from Université Laval. His research focuses on liquid biopsy technologies, particularly extracellular vesicles (EVs) and the information they contain to predict immunotherapy response and provide strategies to overcome immune resistance in cancer. Dr. Ouellette leads multiple projects applying multi-omics to liquid biopsy and using AI to improve cancer outcomes. He has been actively involved in various advisory boards and committees and has also served in leadership roles with organizations such as the New Brunswick Health Research Foundation, the New Brunswick Innovation Foundation, and Prostate Cancer Canada's Research Advisory Board.

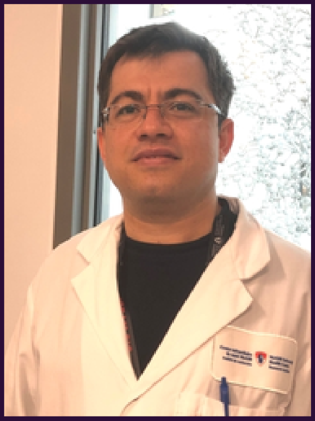

Dr. Orçun Haçariz
McGill University Health Centre, QC

Orçun Haçariz completed his Ph.D. at McGill University. where he focused on multi-omics computation to elucidate complex host-microbiome interactions and also performed bench laboratory techniques using bacterial species and C. elegans as biological model systems. Dr. Haçariz joined Dr. Brodt's Lab at the Research Institute of the McGill University Health Centre in April, 2022, to undertake a postdoctoral fellowship during which he uncovered IGF-2 mRNA binding protein (IMP1)-regulated mediators of PDAC initiation using RNAseq, an AI-based analysis (convolutional neural network for ex-vivo imaging) and plasma exosome analysis for biomarker discovery. Dr. Haçariz gained deep knowledge of the biology of liver metastasis and acquired important skillsets in the study of cancer progression and metastasis. He studied the role of estrogen receptor (ER) signaling and liver immune cells in this context. To date, his work has resulted in 22 scientific articles (12 as first author, 1 as co-first author and 9 as co-author), which have been cited 1477 times.

Unlocking New Horizons: Pancreatic Cancer Clinical Trials and Advances Across Canada

Dr. Saman Maleki
University of Western Ontario

Dr. Sharlene Gill
BC Cancer

PRESENTATION SUMMARY:
Explore the current landscape of pancreatic cancer clinical trials in Canada, emphasizing promising immunotherapy and targeted treatment approaches. This session highlights cutting-edge research demonstrating improved patient outcomes and expanding innovative options for Canadians battling this challenging disease.

LEARNING OBJECTIVES:
By the end of this session, learners will be able to:

- Understand how genomic profiling enables personalized pancreatic cancer treatments by identifying tumor subtypes and genetic markers to guide therapy decisions in clinical trials.
- Summarize emerging immunotherapy approaches, including oncolytic viruses and personalized mRNA vaccines, designed to enhance the immune response against pancreatic cancer cells.
- Evaluate targeted therapies focusing on genetic mutations like KRAS and BRCA, improving treatment efficacy and patient outcomes in pancreatic cancer clinical trials.

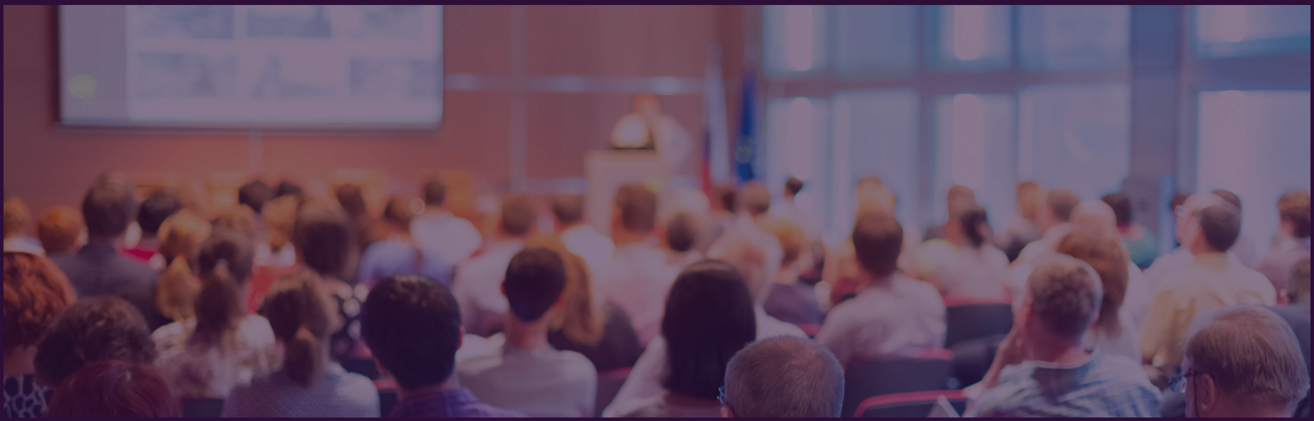

Unlocking New Horizons: Pancreatic Cancer Clinical Trials and Advances Across Canada

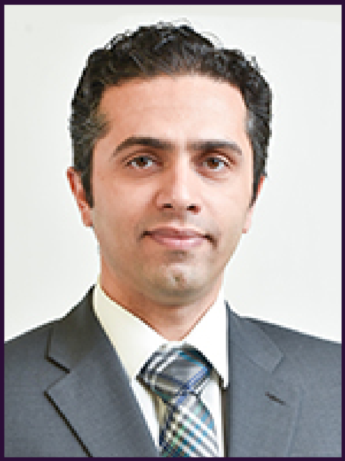

Dr. Saman Maleki
University of Western Ontario

Dr. Saman Maleki is an Associate Professor at the Departments of Pathology and Laboratory Medicine and Oncology at Western University, and a translational immuno-oncology scientist at the London Health Sciences Research Institute (LHSCRI) and the Ontario Institute for Cancer Research in Canada. His research has focused on developing novel strategies to sensitize hard-to-treat cancers to immunotherapy with immune checkpoint inhibitors. He has pioneered clinical trials that, for the first time in Canada and internationally, combine fecal microbiota transplantation (FMT) from healthy donors with immune checkpoint inhibitors in cancer patients—an advancement over previous approaches using patient donors in refractory settings. Dr. Maleki has led the development of multiple phase I/II trials in advanced pancreatic cancer, melanoma, renal cell carcinoma, and lung cancer, serving as co-PI and overseeing protocol development, funding acquisition, and all translational aspects of these trials. In addition to interventional trials, Dr. Maleki leads observational studies examining gut microbiome changes in pancreatic cancer and melanoma patients during treatment.

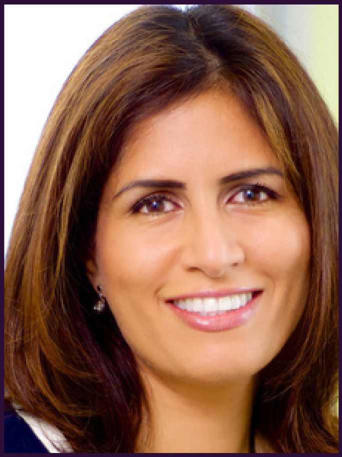

Dr. Sharlene Gill
BC Cancer

Dr. Sharlene Gill is a medical oncologist specializing in gastrointestinal malignancies at BC Cancer – Vancouver. She received a BSc. in Pharmacy, and MD from the University of British Columbia in 1996 followed by residencies in Internal Medicine and Medical Oncology, subsequently completing a fellowship in Gl Oncology at the Mayo Clinic (Rochester, MN) and a Masters of Public Health from the Harvard School of Public Health (Boston, MA) before returning to Vancouver. She is presently Professor of Medicine at the University of British Columbia. In 2017, she completed an MBA from the Kenan-Flagler School of Business at the University of North Carolina. Her areas of clinical expertise are in colorectal, pancreatic and hepatobiliary cancers. She is actively engaged in education and clinical research, with over 100 peer-reviewed publications and book chapters to her credit.

Neoadjuvant Therapy: Transforming the Surgical Landscape of Pancreatic Cancer

Dr. Pablo Serrano

Dr. Beatriz Antón Pascual

PRESENTATION SUMMARY:
Explore the latest advancements in neoadjuvant therapies for resectable pancreatic cancer, focusing on strategies to shrink tumors before surgery. This session highlights clinical trial data, multidisciplinary approaches, and emerging treatment protocols aimed at improving surgical outcomes and long-term survival for patients facing this aggressive disease.

LEARNING OBJECTIVES:
By the end of this session, learners will be able to:

- Understand the role of neoadjuvant therapy in improving surgical outcomes for pancreatic cancer patients.
- Discuss the role of novel (e.g. targeted and immunotherapies) in the neoadjuvant setting.
- Implement multidisciplinary approaches to optimize pre-surgical pancreatic cancer care.

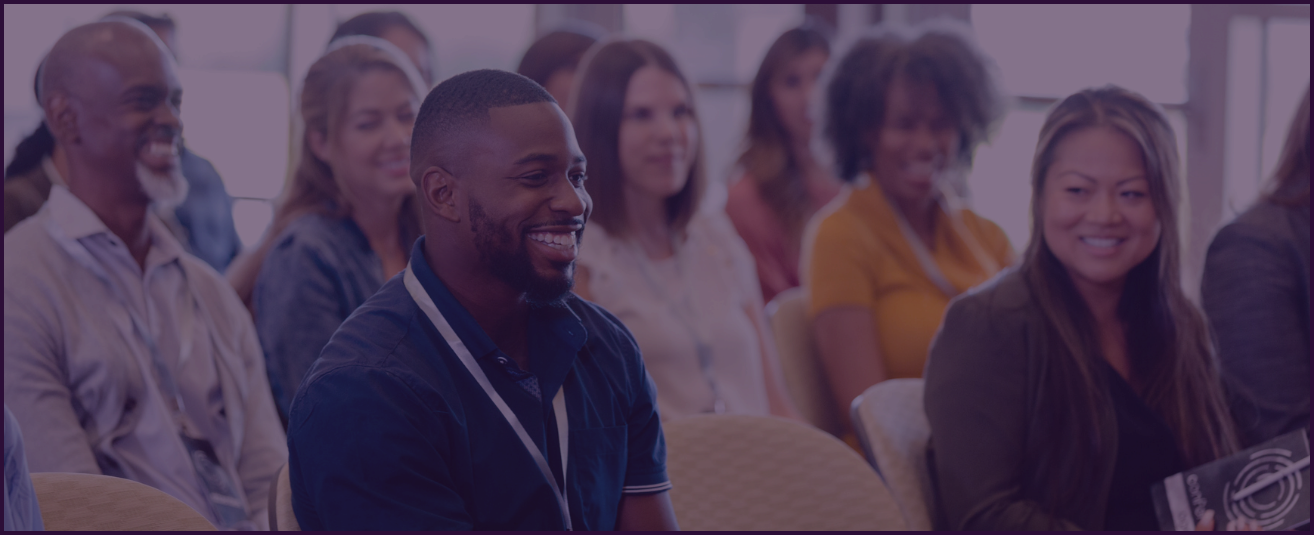

Neoadjuvant Therapy: Transforming the Surgical Landscape of Pancreatic Cancer

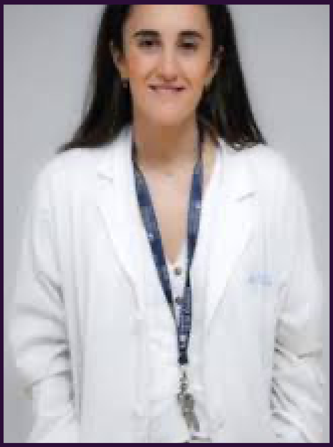

Dr. Beatriz Antón Pascual
Hamilton Health Sciences

Beatriz Antón, MD, is a medical oncologist specializing in gastrointestinal and neuroendocrine tumors. She has earned two master's degrees: one in Oncology Research from Universidad CEU San Pablo (2018) and another in Molecular Oncology from the Spanish National Cancer Research Center (2019). In September 2020, Dr. Antón joined the Gastrointestinal and Neuroendocrine Tumor Oncology Unit led by Dr. García-Carbonero at Hospital Universitario. Since then, she has been actively involved in patient care, clinical trials, and multiple clinical and translational research.
Beatriz is currently pursuing a PhD at Universidad Complutense de Madrid, where she is integrating multi-omic approaches and digital spatial profiling to characterize the tumor immune microenvironment. Most recently, she has started a fellowship at Princess Margaret Cancer Centre in Toronto where she is pursuing advanced training in gastrointestinal tumors.

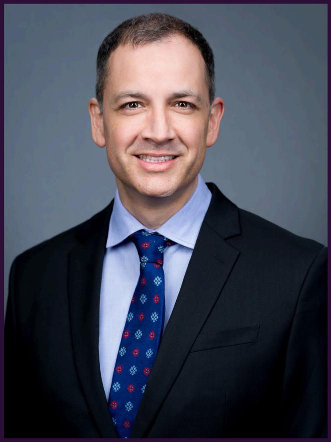

Dr. Pablo Serrano
Hamilton Health Sciences

Pablo E. Serrano is an Associate Professor of Surgery at McMaster University. He joined the Department of Surgery in 2014 after finishing his fellowship in Hepatobiliary Surgical Oncology at the University of Toronto. He completed his General Surgery Residency at the University of Toledo, Ohio where he also completed a Master of Science in Molecular Biology and a Master of Public Health. He completed his PhD in Health Research Methods at McMaster University in 2022. He is an active researcher and scientist at the Escarpment Cancer Research Institute and has led several trials within the Ontario Clinical Oncology Group, an academic Clinical Trials Group based at McMaster University. He has been the recipient of several research grants and is currently the principal investigator of multi-institutional nation-wide multidisciplinary clinical trials. He is also an Associate Member of the Centre for Discovery in Cancer Research at McMaster University, focusing on comprehensive translational studies that inform the rational development of combinational therapies for cancer treatment.

Proven Models, National Impact: Expanding Patient-Centered Pancreatic Cancer Care

Dr. Ken Leslie
Baker Centre for Pancreatic Cancer, ON

Dr. Erica Tsang
Princess Margaret Cancer Centre, ON

PRESENTATION SUMMARY:

This session presents the McCain and Baker Centres as proven models for improving pancreatic cancer survival and quality of life. It explores how adopting these patient-centered, multidisciplinary approaches across provinces offers a vital opportunity to enhance care coordination, support services, and outcomes for patients nationwide.

LEARNING OBJECTIVES:

By the end of this session, learners will be able to:

- Understand the key components of the McCain and Baker Centres' patient-centered care models and their impact on survival and quality of life.
- Identify the challenges and opportunities in adapting these models to other provincial healthcare systems.
- Propose strategies for scaling multidisciplinary, integrated care across Canada.

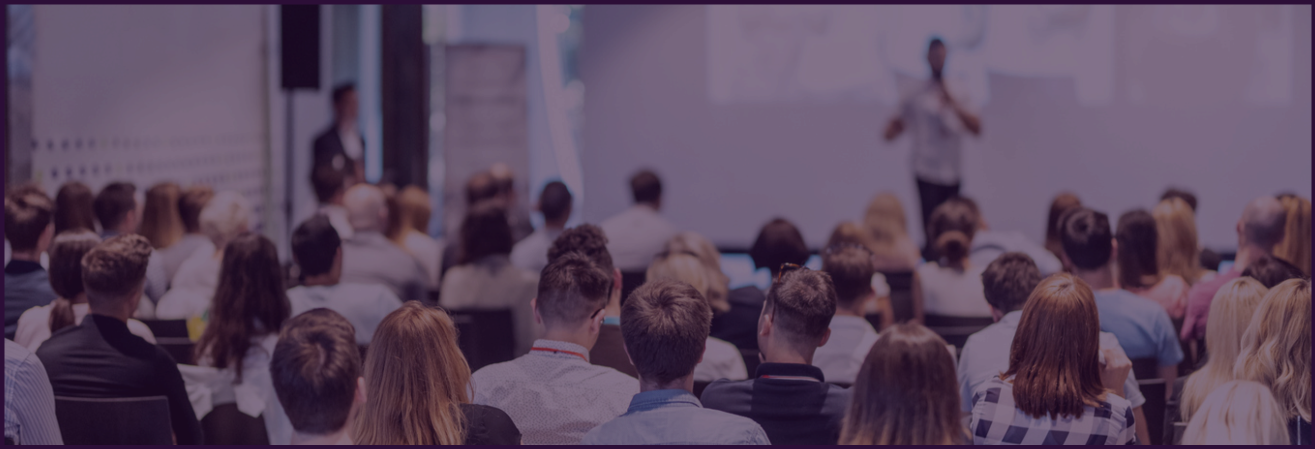

Proven Models, National Impact: Expanding Patient-Centered Pancreatic Cancer Care

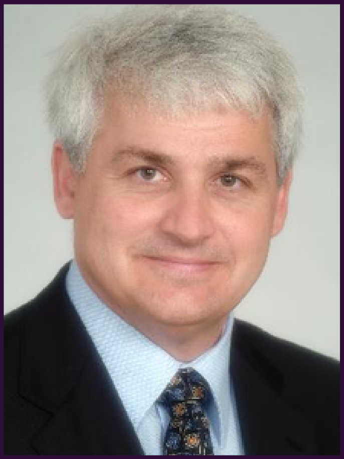

Dr. Ken Leslie
Baker Centre for Pancreatic Cancer, ON

Dr. Leslie completed his medical school and residency training at Western University, followed by a fellowship at the Lahey Clinic in Burlington, Massachusetts. He earned a Masters of Health Professions Education at the University of Illinois at Chicago. Dr. Leslie was appointed as Assistant Professor in the Department of Surgery at Western University in 1995 and promoted to Associate Professor in 2006. Dr. Leslie is a strong contributor to educational and leadership activities in general surgery. He has held appointments as Director of Core Surgery, Director of Surgical Education, Site Chief of General Surgery Victoria Campus and Program Director for General Surgery. He created the postgraduate Introductory Surgery Course in 1996, the Office of Surgical Education in 2000 and the ACCESS program in 2010. Dr. Leslie is an acclaimed teacher and role model, having won many teaching awards of the past few years. In July 2012 Dr. Leslie assumed leadership of the Division of General Surgery and his mission was to produce a division that leads the country in Surgical Education, Research, Surgical Innovation and High Quality of Fiscally Responsible Patient Care. Dr. Leslie's research interests are in surgical education and hepatobiliary pancreatic surgery.

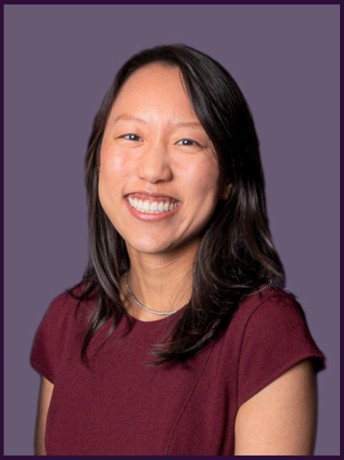

Dr. Erica Tsang
Princess Margaret Cancer Centre, ON

Dr. Erica Tsang is a GI medical oncologist at the Princess Margaret Cancer Centre and an Assistant Professor at the University of Toronto. She completed her Internal Medicine and Medical Oncology training at the University of British Columbia, followed by further training in GI and early phase oncology at the University of California San Francisco. Dr. Tsang also completed a Masters of Public Health at Harvard University. Her research interests involve genomics and clinical trials, with a focus on pancreatic cancer.

Innovations Unveiled: Poster Presentations by Rising Scientists

PRESENTATION SUMMARY:

Explore a diverse collection of innovative research showcased through poster presentations by residents, PhD candidates, and Master's students. This session highlights emerging discoveries and cutting-edge studies in pancreatic cancer, offering a unique opportunity to engage directly with the next generation of researchers and learn about their impactful work.

LEARNING OBJECTIVES:

By the end of this session, learners will be able to:

- Describe recent research focuses and findings from residents and graduate students in pancreatic cancer.
- Engage with emerging scientists to understand current trends and challenges in pancreatic cancer research.
- Gain insights into diverse methodologies and their potential impact on future pancreatic cancer treatments and care.

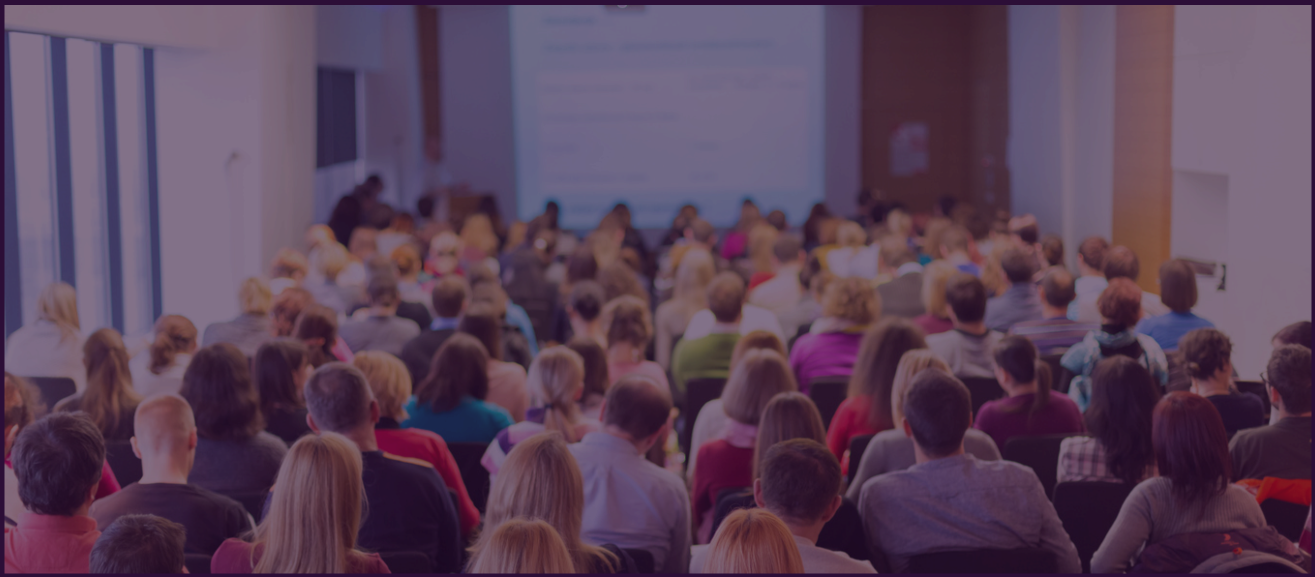

Not Optional: Making Nutrition and PERT Standard in Treatment Pathways

Dr. Popi Kasvis
McGill University Health Centre, QC

PRESENTATION SUMMARY:

This session unveils the new national PERT guidelines for healthcare professionals—recommended at the 2023 National Pancreas Conference. Recognizing nutrition and PERT as critical components of pancreatic cancer care, the guidelines aim to standardize practice, improve outcomes, and support clinicians with tools like the updated PERT Calculator 2.0 for precise dosing.

LEARNING OBJECTIVES:

By the end of this session, learners will be able to:

- Summarize the newly unveiled national PERT guidelines and their role in standardizing pancreatic cancer care across Canada.
- Recognize the clinical impact of nutrition and PERT when integrated alongside traditional treatments like chemotherapy.
- Apply the PERT Calculator 2.0 for accurate, individualized enzyme dosing in practice.

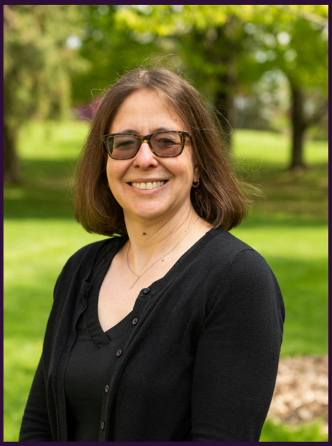

Dr. Popi Kasvis
McGill University Health Centre, QC

Popi Kasvis is a Registered Dietitian and Faculty Lecturer at the School of Human Nutrition at McGill University. Her clinical experience in supportive cancer care and cancer rehabilitation has regularly exposed her to patients with pancreatic cancer. It was within the Cancer Rehabilitation Program at the McGill University Health Centre that she developed her expertise in providing dietary assessment, counselling, and pancreatic enzyme replacement therapy to patients with pancreatic cancer. Her doctoral research focused on how dietary interventions may help improve the health-related quality of life in individuals awaiting pancreatic resection. Popi continues to advocate for improved treatment of pancreatic enzyme insufficiency through her work on the Medical Advisory Committee of Craig's Cause.

From Protocol to Progress: The Therapies Transforming Survival and Quality of Life

Dr. Stephen Welch
London Health Sciences Centre

PRESENTATION SUMMARY:

This session reviews the current national standards for pancreatic cancer surgery and chemotherapy, highlighting the most effective protocols improving survival and quality of life. Experts will examine evidence-based approaches, including neoadjuvant therapy and combination chemotherapy regimens, that show the greatest promise for advancing patient outcomes across Canada.

LEARNING OBJECTIVES:

By the end of this session, learners will be able to:

- Understand current national standards for pancreatic cancer surgery and chemotherapy focused on improving survival and quality of life.
- Discuss evidence supporting combination chemotherapy regimens in enhancing patient outcomes.
- Describe and apply best practices for integrating surgical and systemic treatments within multidisciplinary care models.

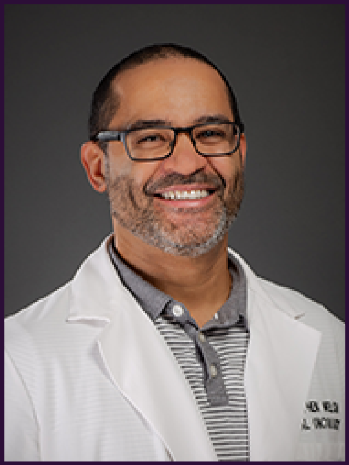

Dr. Stephen Welch
London Health Sciences Centre

Dr. Stephen Welch is a Medical Oncologist at the London Health Sciences Center and Director of the Baker Center for Pancreatic Cancer. In this session, he will review the current national standards for pancreatic cancer surgery and chemotherapy, highlighting the most effective protocols improving survival and quality of life. Dr. Welch will examine evidence-based approaches, including neoadjuvant therapy and combination chemotherapy regimens, that show the greatest promise for advancing patient outcomes across Canada.

Next Steps: Driving Impact and Innovation for Pancreatic Cancer Care

Stefanie Condon-Oldreive
Craig's Cause Pancreatic Cancer Society

PRESENTATION SUMMARY:

This final discussion brings together insights from all conference sessions to chart a strategic path forward for Craig's Cause Pancreatic Cancer Society and other advocacy organizations. Experts and stakeholders will discuss actionable steps to advance research, advocacy, patient support, and care innovation—driving meaningful improvements in pancreatic cancer patient outcomes nationwide.

LEARNING OBJECTIVES:

By the end of this session, learners will be able to:

- Synthesize key insights from the conference to identify priorities for advancing pancreatic cancer care and research.
- Explore strategic roles for advocacy organizations towards improved funding and patient support initiatives.
- Apply action plans to foster collaboration and innovation to improve patient outcomes nationally.

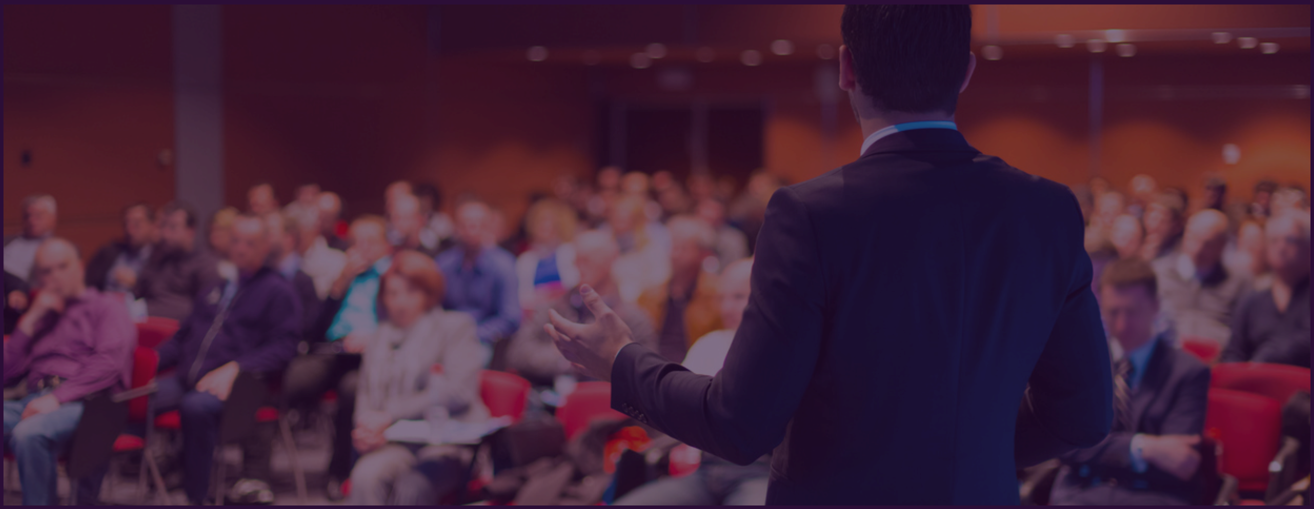

Next Steps: Driving Impact and Innovation for Pancreatic Cancer Care

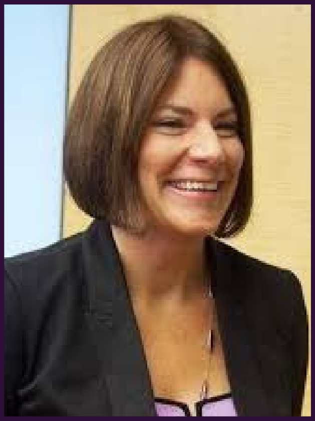

Stefanie Condon-Oldreive
Craig's Cause Pancreatic Cancer Society

Stefanie Condon-Oldreive has been an elementary school teacher with the Halifax Regional Municipality for well over 20 years. In 2006, she established Craig's Cause Pancreatic Cancer Society after her father, Craig Condon, was diagnosed with pancreatic cancer and passed away just 8 weeks later at the age of 63. Stefanie serves as the Founder and Director of Craig's Cause. She is involved in all areas of the Society and has brought in millions of dollars to help pancreatic cancer patients and their families through research as well as various programs such as Support4Caregivers™, Peers with Pancreatic Cancer™, and Patient Financial Support Grants™. In 2021, Stefanie worked with the Canadian Association of General Surgeons to hold the first and only national accredited conference dedicated to pancreatic cancer, the National Pancreas Conference. This event brought healthcare professionals together from across Canada and abroad, to learn, discuss, and collaborate in the field of pancreatic cancer. To help bring awareness, Stefanie has led her team in hosting Kicking PancreAS™ 5K across the country. The event is a fun run/walk for all ages and abilities that shines some light on a dark subject. She spends time fighting for better care for pancreatic cancer patients and doing the biggest job of all, supporting patients and caregivers one-on-one.

The organizers of the National Pancreas Conference would like to thank the following sponsor and partner. With your help, we are working towards a world where surviving pancreatic cancer is expected.

VIATRIS™

NOTES

Craig's Cause Pancreatic Cancer Society
P.O. Box 8561
Halifax, N.S. B3K 5M3
1.877.212.9582
www.craigscause.ca
info@craigscause.ca

#CHANGETHEOUTCOME
